# Biaxial Strain Transfer in Monolayer MoS_2_ and WSe_2_ Transistor Structures

**DOI:** 10.1021/acsami.4c07216

**Published:** 2024-09-03

**Authors:** Antonios Michail, Jerry A. Yang, Kyriakos Filintoglou, Nikolaos Balakeras, Crystal Alicia Nattoo, Connor Scott Bailey, Alwin Daus, John Parthenios, Eric Pop, Konstantinos Papagelis

**Affiliations:** † Department of Physics, University of Patras, Patras 26504, Greece; ‡ Institute of Chemical Engineering Sciences, Foundation for Research and Technology Hellas (FORTH − ICE/HT), Patras 26504, Greece; § Department of Electrical Engineering, Stanford University, Stanford, California 94305, United States; ∥ School of Physics, Department of Solid State Physics, Aristotle University of Thessaloniki, Thessaloniki 54124, Greece; ⊥ Department of Microsystems Engineering, University of Freiburg, Freiburg 79110, Germany; # Department of Materials Science, Stanford University, Stanford, California 94305, United States; ∇ Precourt Institute for Energy, Stanford University, Stanford, California 94305, United States

**Keywords:** biaxial strain, 2D electronics, flexible transistors, strain transfer, Raman spectroscopy

## Abstract

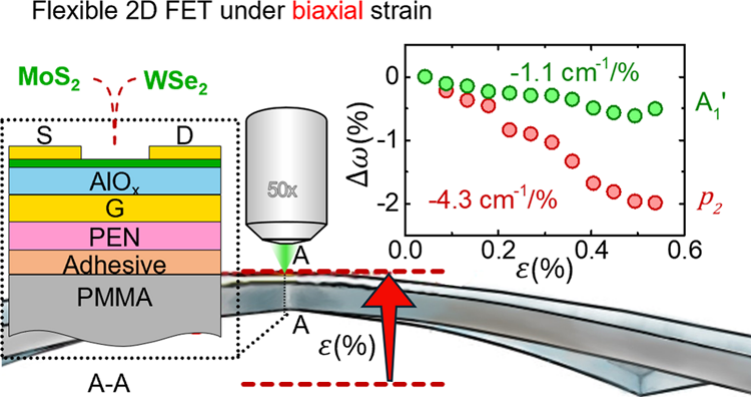

Monolayer transition metal dichalcogenides are intensely explored as active materials in 2D material-based devices due to their potential to overcome device size limitations, sub-nanometric thickness, and robust mechanical properties. Considering their large band gap sensitivity to mechanical strain, single-layered TMDs are well-suited for strain-engineered devices. While the impact of various types of mechanical strain on the properties of a variety of TMDs has been studied in the past, TMD-based devices have rarely been studied under mechanical deformations, with uniaxial strain being the most common one. Biaxial strain on the other hand, which is an important mode of deformation, remains scarcely studied as far as 2D material devices are concerned. Here, we study the strain transfer efficiency in MoS_2_- and WSe_2_-based flexible transistor structures under biaxial deformation. Utilizing Raman spectroscopy, we identify that strains as high as 0.55% can be efficiently and homogeneously transferred from the substrate to the material in the transistor channel. In particular, for the WSe_2_ transistors, we capture the strain dependence of the higher-order Raman modes and show that they are up to five times more sensitive compared to the first-order ones. Our work demonstrates Raman spectroscopy as a nondestructive probe for strain detection in 2D material-based flexible electronics and deepens our understanding of the strain transfer effects on 2D TMD devices.

## Introduction

Two-dimensional (2D) semiconductors have attracted significant interest in recent years due to their unique electronic and optical properties arising from their low dimensionality.^1−6^ One class of 2D semiconductors, transition metal dichalcogenides (TMDs), constitute a promising platform for electronic and optical device applications because of their wide band gaps, layered structure, and well-controlled synthesis.^7−9^ Among these, monolayer molybdenum disulfide (1L-MoS_2_) and monolayer tungsten diselenide (1L-WSe_2_), exhibit good electron and hole mobilities, respectively.^10−12^

Strain engineering (i.e., the intentional use of mechanical deformations to modify material properties), has been used effectively in TMDs to induce changes to the electronic band structure,^13,14^ piezoelectric^15,16^ and piezoresistive effects,^17,18^ quasiparticle funneling,^19−21^ or to enhance carrier mobility.^22−24^ Various modes of deformation have been successfully applied in atomically thin materials including uniaxial,^13,25,26^ biaxial,^27−30^ shear^31^ or nonuniform mechanical strain fields^17^ using a variety of techniques. Nevertheless, current literature has primarily studied monolayers that were either free-standing,^30,32^ simply supported^13,25,33^ or polymer-encapsulated.^34,35^ In contrast, flexible 2D-TMDs-based devices typically involve several heterointerfaces that are required to not only withstand the externally applied mechanical strain but also efficiently transfer it to the TMD crystal to leverage strain-induced behavior. For example, locally back-gated field-effect transistors contain multiple interfaces in the substrate-gate-dielectric-channel stack as well as between the channel and electrical contacts. While other works have shown good strain transfer in devices up to ∼1% for uniaxial strain, biaxial strain transfer in flexible TMD-based devices remains unexplored.^23,30,36−40^

In this work, we demonstrate that very high biaxial strain transfer efficiency can be achieved in 2D material-based flexible devices. In particular, for the first time, we subject several MoS_2_ and WSe_2_ monolayer transistors, fabricated on flexible polyethylene napthalate (PEN) substrates, to tensile biaxial strain. Raman spectroscopy is utilized to measure the imposed strain *in situ*. We show that despite the heterogeneity of interfaces occurring in a typical 2D material transistor, biaxial strains up to 0.55% can be efficiently and controllably imposed on the semiconducting TMD layers in the transistor channel. Because the biaxial strain sensitivity of the main Raman modes of 1L-MoS_2_ is well-established,^28,30^ the efficient strain transfer is first verified for several MoS_2_ devices through detailed Raman mapping over the transistor channel. An analysis of the Raman maps obtained in the undeformed state revealed a distribution of residual strain. Upon strain application, the magnitude of strain in the channel systematically increased in a relatively homogeneous manner, indicating efficient biaxial strain transfer. Furthermore, WSe_2_ devices were investigated using 515 and 632 nm excitation, which offer different resonance conditions.^41^ We also probe the strain response of the higher-order Raman modes of WSe_2_ between 350 to 400 cm^–1^ for the first time, finding a 5-fold strain sensitivity compared to the accidentally degenerate first-order *A*_1_^′^/*E*′ Raman modes. This is an important outcome from fundamental as well as applied perspective since higher-order Raman modes (and their strain sensitivity) are intimately coupled to carrier scattering pathways and the electronic and phononic subsystems of the material.^42−44^ Finally, this work highlights the utility of Raman spectroscopy for strain detection in 2D material based flexible electronics and, furthermore, deepens our understanding of the strain transfer effects on 2D TMD devices, with potential implications for engineering transistor performance as it has been done for strained silicon in the past.

## Methods

### Flexible Transistor Fabrication

**Figure 1:**
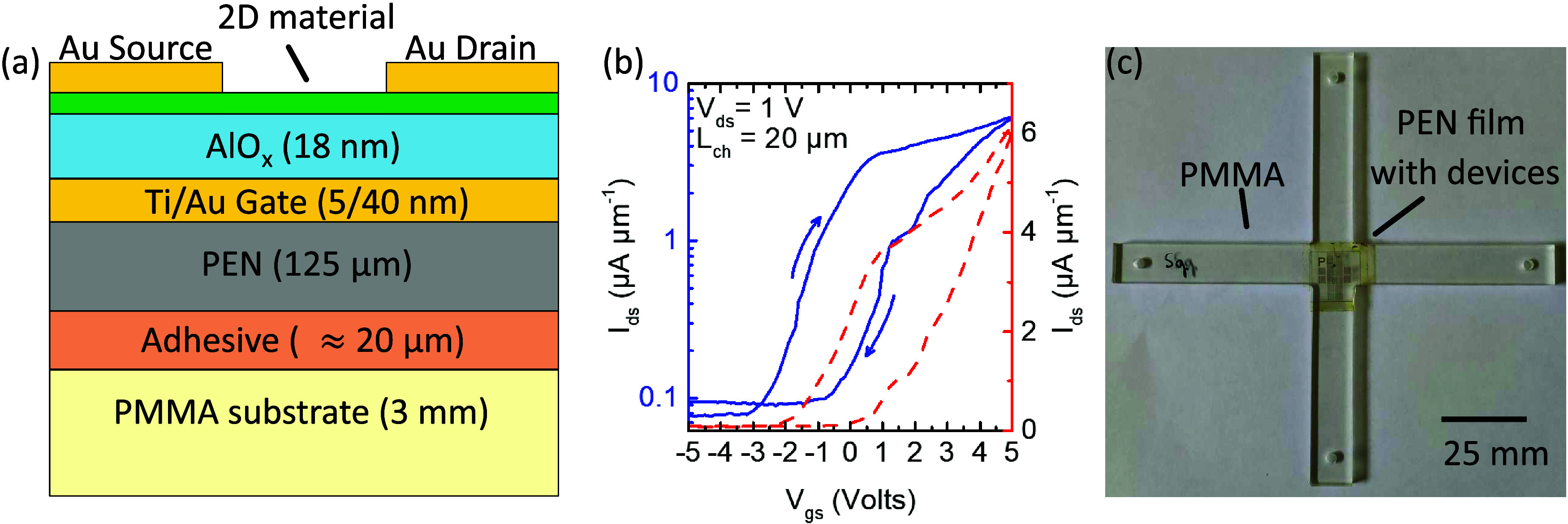
(a) The structure of the transistors that were studied (not-to scale). The thickness of each layer is indicated. (b) Transfer curves *I*_ds_ vs *V*_gs_ for a representative MoS_2_ device with a channel width of 20 μm (*V*_ds_ = 1 V). (c) Image of a PEN film with transistors attached on a PMMA cruciform.

Figure 1a shows the device structure of our 1L MoS_2_ and WSe_2_ field-effect transistors. We first pattern and evaporate 5 nm/40 nm Ti/Au local back gates onto 125-μm thick free-standing polyethylene naphthalate (PEN) substrates, then deposit ≈18 nm Al_2_O_3_ using atomic layer deposition at 130 °C. We grow monolayer MoS_2_ and WSe_2_ onto Si/SiO_2_ substrates with 100 nm thermal oxide via solid-source chemical vapor deposition. We then transfer the TMD onto the PEN substrate using a bilayer PMMA/polystyrene stamp, as described in ref (45), and pattern and evaporate 55 nm Au contacts. Lastly, we define the channel using an O_2_ plasma etch. The flexible PEN film with transistors is attached to a 3 mm thick PMMA cruciform using commercially available cyanoacrylate adhesive (Logo Instant). Section 1 in the Supporting Information discusses in more detail the attachment procedure.

### Application of Biaxial Strain

A custom-made strain jig allows the controlled application of biaxial strain on the cruciform while, at the same time, providing direct optical access to the samples. In particular, a micrometric precision screw (deflection screw) is used to gradually deflect the center of the cruciform in the out-of-plane direction. Since the arms of the cruciform are held in place by constraining pins, the deflection screw bends the cruciform symmetrically out-of-plane.^27−29^ This kind of bending induces tensile biaxial strain at the concave side of the cruciform. More details of the strain jig have been published elsewhere.^29^

### Raman Spectroscopy

All Raman measurements were conducted in the backscattering geometry using three different set-ups. The first setup is a Renishaw InVia Raman spectrometer equipped with a solid state Cobolt Fandango (515 nm) laser and a 2400 grooves/mm grating. The laser beam was focused on the samples using a 50 × (NA: 0.75) objective lens resulting in a spot-size of about 1 μm. Incident power on sample was less than 0.1 mW. High-resolution Raman spectra were recorded using a triple monochromator system (Dilor XY) equipped with a nitrogen-cooled CCD and a 514.5 nm Ar^+^ laser. A 50× long working distance objective was used to focus the beam on the sample, at a power lower than 0.5 mW. Finally, the Raman measurements acquired with the 632.8 nm He–Ne laser line were recorded using a LabRam HR (HORIBA) system equipped with a Peltier-cooled CCD. The laser was focused on the sample with a 50× long working distance objective at a power lower than 0.1 mW.

## Results and Discussion

### MoS_2_ Transistors

Figure 1b shows the forward and backward drain current (*I*_ds_) vs gate voltage (*V*_g_) sweeps of a representative MoS_2_ device. The devices have a constant-current threshold voltage of about −3 V, extracted using the current threshold of 100 pA, consistent with the International Roadmap for Devices and Systems.^46^ We calculate the carrier concentration at *V*_gs_ = 0 V from the *I*_ds_ – *V*_gs_ curves using *n*_2D_ = *C*_ox_·(*V*_gs_*V*_T_), where *C*_ox_ ≈ 350 nF/cm^2^ is the gate oxide capacitance per unit area. This results in a charge density *n*_2D_ ≈ 6.6 × 10^12^ cm^–2^ in the channel region. We note that device performance is suboptimal compared to state-of-the-art 2D material devices. This, however, is attributed mainly to the following three factors. First, the devices were electrically measured in air and were not encapsulated, thus the transistor channel was exposed to ambient conditions. This way water molecules and/or other adsorbates could adsorb in the channel during measurement, increasing the device hysteresis.^47^ Second, contact resistance may contribute to lower ON-current, as Au is known to cause Fermi level pinning near the midgap in MoS_2_ and WSe_2_.^48^ Third, device fabrication involves a wet transfer process, which may cause additional damage to the TMD and introduce PMMA polymer residues onto the TMD surface.^49^ While other works have achieved more optimal contacts with other metals, contact resistance optimization is outside the scope of this work.

After attachment of the flexible transistors onto a PMMA cruciform using a cyanoacrylate adhesive (Figure 1c), biaxial strain is applied on the cruciform (see Methods). Due to interfacial shear forces between PMMA/adhesive and adhesive/PEN film interfaces, this applied strain is then transferred to the PEN film. Note, however, that the strain imparted on the device-side of the PEN film is not necessarily of equal magnitude to the strain that is applied at the cruciform’s top surface in the absence of the PEN film. The ratio between the strain at top of the PEN film, ε_PEN_, to the applied strain on the cruciform, ε_PMMA_, is known as the strain transfer efficiency and depends on the adhesive thickness and its mechanical properties. The strain transfer efficiency from cruciform to the PEN film was assessed using strain gauge sensors for several adhesives. Among the compounds that were tested, cyanoacrylate adhesive (CA) presented an average strain transfer efficiency of 87%, preserved the linearity of applied strains, and formed a thin adhesion layer with a nominal thickness of the order of 20 μm (see Supporting Information Section 1).

**Figure 2:**
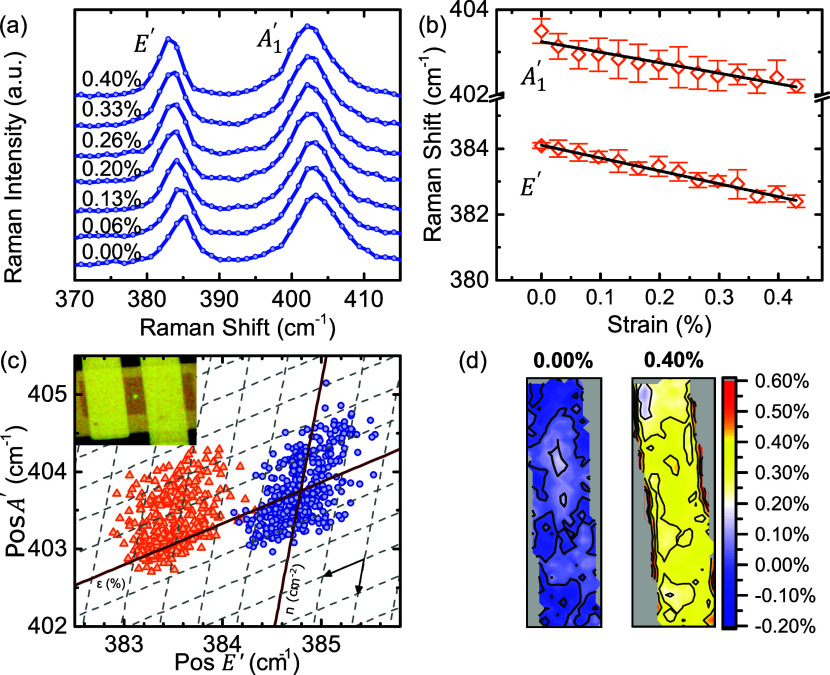
Strain-dependent Raman spectroscopy in an MoS_2_ flexible transistor. (a) Evolution of the Raman spectra at various strain levels. (b) Strain dependence of the *A*_1_^′^ and *E*′ peak frequencies. The error bars indicate the standard deviation of the measurements. (c) Pos (*A*_1_^′^) - Pos (*E*′) plot and corresponding strain - doping axes for Raman maps at zero (blue) and maximum (orange) strain for transistor MT1. The grid size of the ε–*n* axes is 0.1% and 2 × 10^12^ cm^–2^, respectively. The black arrows indicate the positive direction of each axis. Inset: Optical microscope image of the probed device. (d) Contour plots of mechanical strain in the transistor channel region at zero strain (left panel) and maximum applied strain (right panel).

As a first step, MoS_2_ devices were investigated under various levels of biaxial strain using Raman spectroscopy. Four devices were measured in total (labeled MT1 to MT4) all having a nominal channel width of 20 μm and channel length of 5 μm except for device MT4 with a channel length of 7 μm. Considering that the probed devices were located at different positions relative to the center of the cruciform, slightly different strains are imposed at each device which, however, can be determined from the methodology presented in a previous work.^29^ The probed devices were all between 1 up to 2 mm from the center of the cruciform and the corresponding strain level differed less than 5%. Figure 2a shows Raman spectra from the central region of an MoS_2_ transistor channel as a function of applied strain. Both peaks shift to lower frequencies with increasing biaxial strain, with *E*′ being more sensitive to the *A*_1_^′^, (Figure 2b). Importantly, no widening or splitting of the *E*′ peak is observed, as expected for biaxial strain. The dependence of the peak positions for the other transistors that were studied (devices MT1, MT2 and MT4) are shown in Figure S3, and exhibit similar trends. The average slopes for *E*′ and *A*_1_^′^ peaks were determined at −4.3(1) and −2.5(3) cm^–1^/%, respectively. Table 1 summarizes the phonon frequency at zero strain, ω_o_, strain-induced shift rate, , and the corresponding mode Grüneisen parameter, , for all tested MoS_2_ devices. The results are in very good agreement with the reported shift rates and Grüneisen parameters for CVD MoS_2_ monolayers under biaxial tension.^28,30^

**Table 1:** Raman Peak Positions at Zero Strain, Strain-Induced Shift Ratesand Mode Grüneisen Parameters for MoS_2_ Devices

Raman mode	device	**ω**_**O**_ **(cm^–1^)**	**(cm^–1^)/%**	**γ**
***E***_**1**_^**′**^	MT1	384.5	–4.6(2)	0.59(2)
	MT2	384.5	–4.3(1)	0.56(2)
	MT3	384.1	–3.9(1)	0.51(1)
	MT4	384.4	–4.6(1)	0.60(1)
				
***A***_**1**_^**′**^	MT1	403.2	–2.4(3)	0.30(2)
	MT2	403.1	–2.4(3)	0.30(1)
	MT3	403.2	–2.5(2)	0.31(2)
	MT4	402.6	–2.7(3)	0.33(4)

Apart from single point spectra per strain level, detailed Raman maps were collected at zero and maximum applied strains on all four devices. Figure 2c shows the Pos*A*_1_^′^ – Pos*E*′ correlation plot for device MT1 at zero and maximum applied strain as blue circles and orange triangles, respectively. Note that the mean position of the unstrained cluster was used as a reference point for the strain–doping axes. For both cases, the data form clearly distinguished clusters. The unstrained cluster presents an average Pos(*E*′), Pos(*A*_1_^′^) value of 384.8 and 403.8 cm^–1^ with a standard deviation of 0.2 and 0.4 cm^–1^, respectively. On the other hand, at the maximum applied strain of 0.43%, the cluster shifts to a new position with Pos(*E*), Pos(*A*_1_^′^) = 383.4 ± 0.2 and 403.4 ± 0.4 cm^–1^, respectively. Overlaying the strain and doping axes in the correlation plot with reference to the unstrained state, it is found that, at maximum applied strain, the MoS_2_ experiences mainly mechanical strain with only slight change of the doping levels (roughly 2 × 10^12^ cm^–2^). Importantly, the almost uniform shift of the cluster indicates homogeneous strain transfer across the channel region. As shown in the strain contour maps at zero and maximum strain presented in Figure 2d, mechanical strain in the channel region is increased but the initial strain distribution roughly remains unchanged. Specifically, the initial residual strain pattern, imparted to the monolayer due to the transferring and subsequent nanofabrication steps, can also be roughly discerned in the maximum strain contour. This phenomenon is observed in all four transistors studied. The corresponding strain maps and ε*–n* correlation plots for devices MT2, MT3 and MT4 are presented in Supporting Information Figure S4. This is an important result, indicating that mechanical strain can indeed be homogeneously transferred to the transistor channel, despite the complex interfaces and geometry of the transistors.

### WSe_2_ Transistors

Representative *I*_d_ – *V*_g_ transfer curves at zero strain for five WSe_2_ devices are presented in Figure S5 in the Supporting Information. Semiconducting behavior and ambipolarity are observed, confirming that the material quality is sufficient for field-effect transistor operation. Having established that mechanical strain can be transferred efficiently in the channel of an MoS_2_ transistor, attention was shifted to monolayer tungsten diselenide (WSe_2_) devices, because it has hardly been studied under biaxial deformations despite its significance as a potential p-type semiconductor in flexible electronics. Hence, a systematic strain-dependent Raman study was carried out on WSe_2_ transistors having identical structure to the previously studied MoS_2_ devices. Additionally, since the biaxial strain response of WSe_2_ is not known, contrary to other TMDs such as MoS_2_^28,30^ two samples having a simpler structure were also tested under strain in order to establish a reference point. The first sample was fabricated by transferring as-grown CVD 1L-WSe_2_ crystals directly onto a PEN film, which was then subsequently bonded to a PMMA cruciform. The second sample was identical to the first one except for an additional 20 nm thick ALD alumina layer deposited onto the PEN film prior to transferring the WSe_2_ monolayers.

Representative Raman spectra of these two samples are compared to a spectrum obtained from the channel region of a 1L-WSe_2_ transistor in Figure S6 in the Supporting Information. It is evident that the spectra from the reference samples (WSe_2_/PEN and WSe_2_/AlO_*x*_/PEN) exhibit strong background luminescence originating from the PEN film. In contrast, the spectrum from the 1L-WSe_2_ in the transistor channel presents a very low background intensity. The background luminescence and prominent Raman bands around 520 and 780 cm^–1^ are attributed to the excitation of the PEN substrate. Unfortunately, their presence partially obscures accurate measurement of the WSe_2_ Raman modes apart from the very prominent peak around 250 cm^–1^. In contrast, the 40 nm thick Au layer in the gate electrode of the transistors effectively blocks the excitation beam from reaching the PEN film substrate, as its thickness is roughly twice as large as the penetration depth of λ = 515 nm radiation on Au (≈20 nm).^50^ This screening effect not only allows for the recording of excellent quality spectra free from extrinsic influences, but also enables the detection of additional Raman modes of WSe_2_ in the 300 to 440 cm^–1^ spectral region (inset of Figure S6). Note that similar effects have been have been observed for FETs fabricated on polyimide instead of PEN.^12^ This indicates that using an ultrathin film of gold (or any other noble metal) in a similar manner has the potential to enable the collection of high-quality spectra. Finally, the strain dependence of the *A*_1_^′^ and 2*LA* modes for the reference samples is presented in Supporting Information Figure S7. The shift rate of the *A*_1_^′^ mode was determined at −1.0(2) and −1.7(3) cm^–1^/%, while the shift rate of the 2*LA* mode was determined at −1.5(3) and −1.8(4) cm^–1^/%, for the WSe_2_/PEN and WSe_2_/AlO_*x*_/PEN sample, respectively.

**Figure 3:**
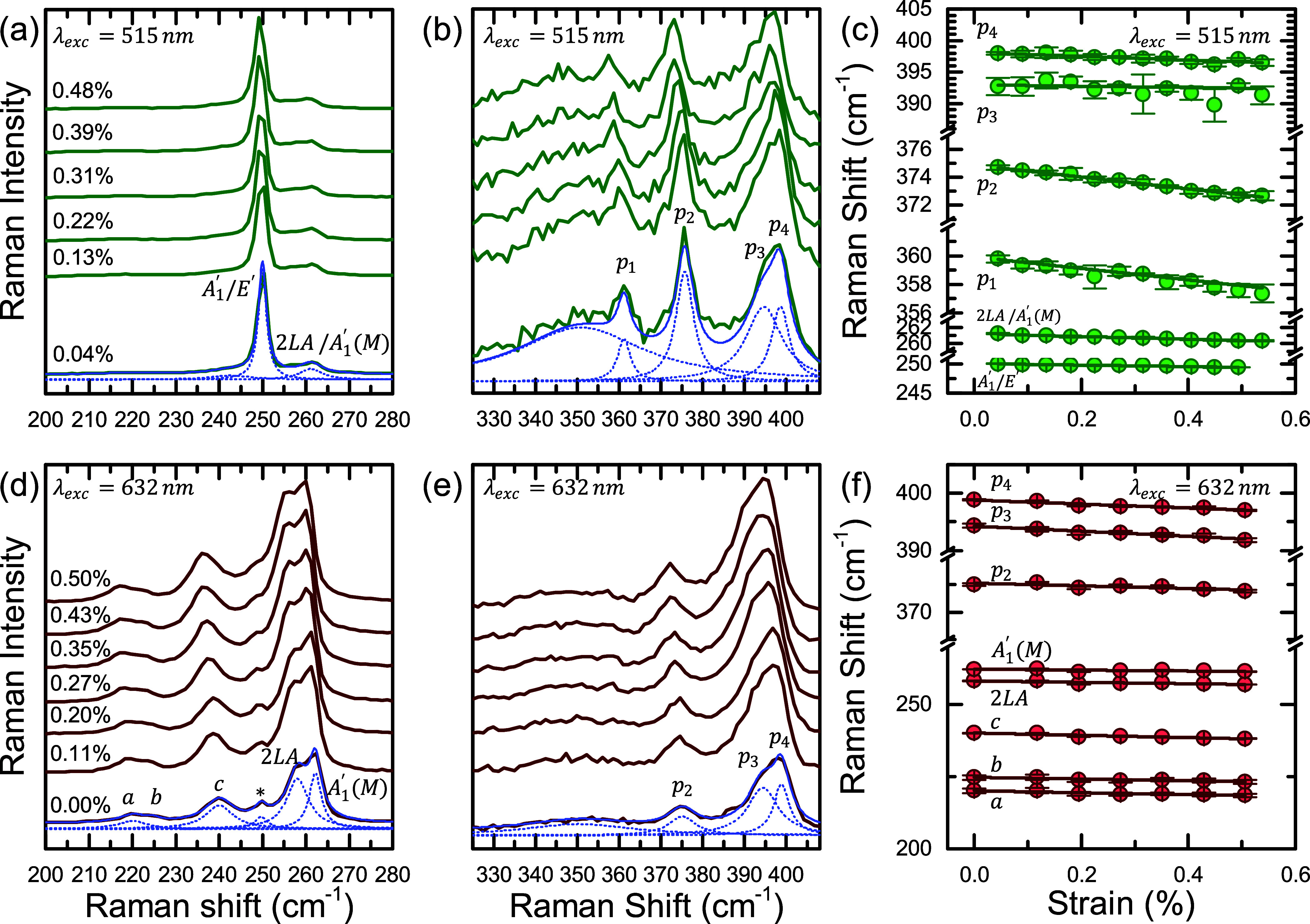
Evolution of the Raman spectrum of WSe_2_ with strain and strain dependence of the peak frequencies for 515 nm excitation in panels (a)–(c), and 632.8 nm excitation in (d)-(f). In (a), (b), (d) and (e) dashed blue curves are the Lorentzian components fitted to the spectrum. The solid lines in (c), (f) are the best fit curves. The error bars indicate the standard deviation of the measurements.

Figure 3a,b show two spectral regions from representative Raman spectra of a WSe_2_ transistor, obtained with λ = 515 nm excitation. The spectra were collected at increasing levels of tensile biaxial strain reaching up to about 0.5%. In the lower frequency region (Figure 3a), the most prominent feature is located around 249 cm^–1^ and is attributed to the out-of-plane, *A*_1_^′^, and in-plane, *E*′, Raman-active zone-center optical phonons. The accidental degeneracy of the *A*_1_^′^ and *E*′ phonon frequencies, which occurs only in the monolayer limit,^51^ can make determining strain from Raman peaks challenging due to the much lower sensitivity of the *A*_1_^′^ mode compared to the *E*′ mode. An alternative approach is to employ polarization-resolved Raman scattering (linear or circular), which enables the selection of only one of the *E*′ and *A*_1_^′^ peaks depending on the polarization configuration.^52,53^ Note, however, that the almost identical frequency and similar widths of the *E*′ and *A*_1_^′^ modes for the monolayer WSe_2_ complicates the peak deconvolution and assignment process.

The comparatively weak feature around 260 cm^–1^ is known as the 2*LA* peak and is assigned to second-order two-phonon scattering involving two longitudinal acoustic (*LA*) phonons from the Brillouin zone boundary. Note that a closer inspection of the Raman line shape of the 2*LA* feature, as recorded with 515 nm excitation, hints to the contribution of at least two components. Indeed, the line shape of the 2*LA* mode can potentially include scattering from at least two critical points of the *LA* branch, specifically, a local maximum at the *M* point and a saddle point located along the *MK* high symmetry line.^54^ The frequency of the *LA* branch at the aforementioned critical points is around 130 cm^–1^, indicating that the 2*LA* overtones are expected around 260 cm^–1^. This is consistent with the detected broad and asymmetric feature detected close to 260 cm^–1^ as shown in Figure S8. However, in at least one work, the high frequency component of this feature close to ≈263 cm^–1^ is attributed to the defect-activated *A*_1_^′^(*M*).^55^ This is reasonable since there is a large contribution to the phonon density of states near this frequency due to the *A*_1_^′^ branch. It must nevertheless be stressed that while critical point analysis can provide interesting insights, a proper determination of the line shape of higher-order modes can be vastly more complex and demands a more rigorous analysis which is beyond the scope of this work.^43^ Note that in the spectra obtained with 515 nm excitation, this feature was fitted with only one Lorentzian component since attempts to fit this feature with two Lorentzians resulted in unstable fits. However, an additional experiment with 514.5 nm excitation and a higher resolution spectrometer resolved both 2*LA* and *A*_1_^′^(*M*) peaks (see Supporting Information Figure S9).

In the higher frequency region between 300 and 440 cm^–1^ (Figure 3b), a bundle of five Raman peaks is detected. Since the highest phonon frequency in 1L-WSe_2_ lies close to 300 cm^–1^, these peaks can only be assigned to higher-order scattering processes involving more than one phonon, i.e., overtones or phonon combinations. The exact assignment of those peaks is not yet clearly established. They are so far attributed to multiphonon combination or difference processes between optical and acoustic phonons.^51,55^ For this reason, the four high-order sharp peaks that were monitored successfully under strain are labeled as *p*_1_ to *p*_4_, as shown in Figure 3b. The lowest frequency broader peak around 350 cm^–1^ is not discussed further in this study because large uncertainties occurred in determining its position during curve fitting. We note that while for peak *p*_1_ the signal-to-noise ratio is rather low compared to other higher-order peaks, the peak position can nevertheless be extracted satisfactorily by multiple Lorentzian fitting as shown in Figure 3c.

Figure 3c shows the strain dependence of the frequencies of all six Raman bands that were adequately monitored in the spectra from a representative device. With increasing tensile strain, all recorded peaks are found to shift to lower frequencies. From each of the five WSe_2_ transistors that were investigated with 515 nm excitation, up to four spectra were collected from the central region of the channel of each device at each strain level. The points in Figure 3c show the mean peak position and the error bars indicate the standard deviation of the measured frequencies. The determined shift rates for all Raman peaks and investigated devices (labeled WT1 to WT4) are summarized in Table 2. It is stressed that the *A*_1_^′^ mode presents an average shift rate of −1.3(1) cm^–1^/% which compares satisfactorily with the obtained shift rates of −1.0(2) and −1.8(3) cm^–1^/%, respectively, for the WSe_2_/PEN and WSe_2_/AlO_*x*_/PEN reference samples presented earlier. Furthermore, the higher-order peaks (*p*_1_ to *p*_4_) are found to be up to five times more sensitive to strain compared to the *A*_1_^′^ mode and, thus, appear as more appealing strain indicator compared to *E*′/*A*_1_^′^. Importantly, these results are in very good agreement with the higher resolution Raman experiment presented in the Supporting Information section 7.

**Table 2:** Raman Peak Positions, Strain-Induced Shift Rates and Grüneisen Parameters for WSe_2_ Devices Excited with λ_exc_ = 515 nm

	***A***_**1**_^**′**^			***2LA***			***p***_**1**_		
	**ω**_**o**_**(cm**^**–1**^**)**	**(cm**^**–1**^**/%)**	**γ**	**ω**_**o**_**(cm**^**–1**^**)**	**(cm**^**–1**^**/%)**	**γ**	**ω**_**o**_ (cm^–1^)	**(cm**^**–1**^**/%)**	****γ****
**WT1**	250.0	–1.43(8)	0.28(2)	261.0	–2.33(8)	0.45(2)	359.6	–5.8(4)	0.80(5)
**WT2**	250.0	–1.5(1)	0.30(2)	261.2	–2.0(1)	0.39(2)	359.9	–4.8(5)	0.66(7)
**WT3**	250.0	–1.1(2)	0.22(3)	261.3	–2.2(2)	0.42(2)	360.7	–6.3(4)	0.87(5)
**WT4**	250.1	–1.31(7)	0.26(1)	261.4	–2.40(9)	0.46(2)	360.2	–5.6(4)	0.78(5)
**WT5**	250.1	–1.39(8)	0.28(2)	261.6	–2.9(2)	0.56(3)	360.9	–6.3(5)	0.87(7)

	***p***_**2**_			***p***_**3**_			***p***_**4**_		
	**ω**_**o**_**(cm**^**–1**^**)**	**(cm**^**–1**^**/%)**	**γ**	**ω**_**o**_**(cm**^**–1**^**)**	**(cm**^**–1**^**/%)**	**γ**	**ω**_**o**_**(cm**^**–1**^**)**	**(cm**^**–1**^**/%)**	**γ**
**WT1**	374.5	–4.8(2)	0.65(3)	392.8			397.9	–3.3(9)	0.4(1)
**WT2**	374.9	–5.0(1)	0.67(2)	392.9			398.1	–3.5(6)	0..44(7)
**WT3**	375.4	–4.9(4)	0.65(5)	394.3	–5(1)	0.6(1)	398.6	–3.6(4)	0.45(5)
**WT4**	375.0	–4.3(2)	0.57(3)	393.4	–3.3(5)	0.42(7)	398.4	–3.6(6)	0.45(7)
**WT5**	375.6	–5.2(3)	0.69(4)	394.0	–3.4(9)	0.4(1)	398.8	–4.2(4)	0.53(5)

Two other WSe_2_ devices WT6, WT7 were also studied in a following experiment under λ = 632.8 nm excitation. The evolution of the Raman spectra as a function of strain is presented in Figure 3d,e for the lower and higher frequency regions (as in (a) and (b)), respectively. Due to the different resonance conditions, new bands are now visible in the lower frequency region. Three new peaks are detected around 219, 223, and 239 cm^–1^ and are labeled *a*, *b* and *c*, respectively. The most notable difference, compared to the excitation with green laser (515 nm), is that the 2*LA*/*A*′(*M*) feature is now the most prominent one, with two clearly resolvable components. This time the *A*_1_^′^(Γ) peak, indicated by the “*” symbol, is barely visible between the *c* and 2*LA* peaks. No new peaks were detected in the higher frequency region, and only the *p*_2_ – *p*_4_ peaks were of adequate intensity. Also, in contrast to what is observed with 515 nm excitation, the *p*_3_ and *p*_4_ peaks are now three to four times more intense than *p*_2_.

Note that in multiple-phonon scattering an excited carrier is scattered consecutively by two or more phonons. For excitation energies near the visible spectrum the selection rules relax and require that the wavevectors of the participating phonons sum to zero.^42^ In general, the intensity of higher-order Raman modes is expected to be weaker compared to first-order modes. However, when the intermediate states occupied by the excited carrier are real electronic states of the system instead of virtual states (resonance condition), a significant enhancement of the scattering cross-section for these participating phonons is observed.^43,44^ The process is said to be a single-, double- or multiple-resonance, depending on how many of the intermediate states satisfy the resonance condition. In some cases, the higher-order modes can be of comparable intensity to the first-order modes. An excellent manifestation of resonant Raman effects is the large enhancement of the intensity of the 2*LA* mode in semiconducting TMDs. For example, for 632.8 nm excitation in WSe_2_ (see Figure 3), or for WS_2_ monolayers with 514.5 nm excitation, as shown in ref (56). It must be noted that in a resonance Raman scattering process the electronic and phononic systems of the material are intimately coupled.^42^ If the matrix element for the process is not vanishing due other reasons (e.g., symmetry considerations), the scattering cross-section is strongly enhanced only for those phonons whose energy and wavevectors “connect” two (or more) real electronic states within the same or neighboring conduction or valence band valleys. For example, in semiconducting TMDs such as WSe_2_, a common intervalley scattering pathway occurs between the conduction band valleys located at the *K*/*K*′ point of the Brillouin zone that are connected by phonons with wavevectors near *K*.^43,57^ Thus, under appropriate resonance conditions, certain phonons near the *K* point can contribute to the recorded Raman spectrum. Here we note that mechanical strain significantly impacts both the electronic band as well as the phonon band structure of the material.^13,29,30^ Hence, the strain-induced frequency shift of higher-order modes is ultimately influenced by both contributions. However, as mentioned earlier, the exact calculation of the Raman cross-section is a much more complicated and cumbersome process, depending strongly on the specific electronic and phonon dispersions that are used in the calculations.^58−60^ Such a detailed treatment of resonance Raman effects in WSe_2_ is out of scope of this work. Nevertheless, the strain dependence of the Raman modes of WSe_2_ presented here, is valuable for future treatments of the phenomenon.

The strain dependence of the detected peak positions is presented in Figure 3f. Again, all peaks were observed to redshift with applied biaxial strain. Table 3 summarizes the determined shiftrates. Importantly, for both devices measured under 632.8 nm excitation, the strain-induced shift rates for most of the detected Raman peaks are similar. The larger uncertainties are mainly due to the lower spectral resolution of the Raman spectrometer for 632.8 nm excitation (see Methods) compared to the 515 nm. For example, the average shift rate for the features assigned to the 2*LA*/*A*_1_^′^ mode with 515 nm excitation, is −2.3(1) cm^–1^, while for 632.8 nm excitation the 2*LA* and *A*_1_^′^(*M*) peaks presented shift rates of −2.4(7) cm^–1^/% and −1.6(7) cm^–1^, respectively. On the other hand, the average shift rate of peak *p*_2_ under 632.8 nm excitation was found to be about −2.6(7) cm^–1^/%, which is lower compared to average shift rate of −4.8(2) cm^–1^/% observed for 515 nm excitation. This is not surprising, considering the significantly different excitation conditions which probe phonons from different regions of the BZ. Overall, peak *p*_2_ under 515 nm excitation seems to be the best compromise between strain sensitivity and simplicity of peak fitting.

**Table 3:** Raman Peak Positions, Strain-Induced Shift Rates and Grüneisen Parameters for WSe_2_ Devices Excited with λ_*exc*_ = 632.8 nm

	**WT6**			**WT7**		
**Raman mode**	**ω**_**o**_**(cm**^**–1**^**)**	(cm^**–1**^**/%)**	**γ**	**ω**_**o**_**(cm**^**–1**^**)**	(cm^**–1**^**/%)**	**γ**
*a*	219.2	–2.9(8)	0.7(2)	220.1	–3.2(8)	0.7(2)
*b*	223.7	–3(1)	0.6(3)	224.7	–3.0(6)	0.7(2)
*c*	239.0	–3.2(6)	0.7(1)	240.0	–4.0(8)	0.8(2)
2*LA*	257.4	–2.4(6)	0.5(1)	258.1	–2.4(8)	0.5(2)
*A*_1_^′^(*M*)	261.7	–1.6(6)	0.3(1)	262.1	–1.5(8)	0.3(2)
*p*_2_	374.8	–2.9(7)	0.4(1)	375.3	–2.4(7)	0.32(9)
*p*_3_	393.0	–3.8(3)	0.50(4)	394.2	–4.4(5)	0.56(7)
*p*_4_	398.0	–3.4(3)	0.42(4)	398.8	–3.4(4)	0.43(6)

The strain-dependent Raman spectroscopy in 1L-MoS_2_ devices provided strong evidence that biaxial strain can be effectively transferred through both the gate electrode and dielectric, as the detected Raman peak positions varied linearly with applied strain and at rates compatible with previously published data.^28^ In particular, the detected strain-induced shift rates of the first-order Raman modes of MoS_2_ are on the high end of the reported values for simply supported CVD MoS_2_. This indicates that the various functional layers of the device (i.e., gate and gate dielectric) are robust in transferring the applied strain from PEN to MoS_2_ (at least up to 0.5%). As such, the strain transfer efficiency from PEN film through the gate electrode, gate oxide and finally the MoS_2_ layer is found to be very close to 100% for the transistor geometries used here. The Young’s modulus of PEN (5.5 GPa) is one to 2 orders of magnitude lower than the moduli of Au, AlO_*x*_ and MoS_2_. This is reminiscent to the reinforcement effect,^61^ where a strain gauge bonded to a low modulus material (substrate) leads to inefficient strain transfer from the substrate to the strain gauge (the transistor in our case).^61^ To explore the mechanics of strain transfer further, we model the adhesive-PEN-TMD transistor stack using an integro-differential equation that captures the strain transfer efficiency in uniaxial loading based on previous work by Stehlin.^62^ For an MoS_2_/AlO_*x*_ (18 nm)/Au (40 nm)/Ti (5 nm) stack supported on a PEN substrate, the calculated strain transfer efficiency for various channel lengths is presented in Figure S11. The strain transfer efficiency as a function of the logarithm of the channel length (*L*) is a typical s-shaped curve with 50 and 90% efficiencies attained at 154 and 1150 nm, respectively. For the 5 μm long channels in the devices studied in this work, the strain transfer efficiency is 98%, justifying that the strain experienced by the 2D materials in the tested devices is essentially equal to the applied strain directly under the gate electrode.

## Conclusions

Biaxial tensile strain can be successfully applied to flexible TMD devices fabricated on thin (125 μm) flexible PEN substrates. Using resistive strain gauges, we determined that the strain transfer from cruciform to the PEN film is efficiently mediated by the thin cyanoacrylate adhesive layer. Notably, this adhesive layer was found to form a mechanically robust interface capable of preserving the linearity of the externally applied strains up to at least 0.7%. Strain dependent Raman measurements were performed in single layer MoS_2_ devices supported on PEN films for strains up to 0.5%. The detected strain-induced Raman peak redshifts for the zone center *E*′ and *A*_1_^′^ modes were found to be in excellent agreement with the corresponding values obtained for simply supported 2D crystals on PMMA. Furthermore, the residual strain distribution, imposed due to device fabrication steps, was determined by Raman mapping in the channel region at zero external strain. A similar Raman mapping at maximum applied strain revealed that the externally applied strain is imposed in a rather homogeneous manner. This strongly suggests that the various interfaces formed between the substrate, the gate, and the gate dielectric are robust, and facilitate transferring of the applied strain all the way to the 2D material in the device channel.

Finally, using two different excitations of 515 and 632 nm, we performed strain-dependent Raman measurements on a set of 1L-WSe_2_ devices and two reference samples with simpler structures (WSe_2_/PEN and WSe_2_/AlO_*x*_/PEN). For the 515 nm excitation, the strain dependence of the first-order *A*_1_^′^/*E*′ Raman bands obtained for the devices is in good agreement with that of the reference samples. Very strong substrate luminescence was observed in the reference samples, which was, however, quenched in all devices due to the presence of the gate electrode (5/40 nm Ti/Au). This enabled us to determine the strain sensitivity of the higher-order Raman peaks of WSe_2_, which is reported for the first time and is found to be up to five times higher compared to the first-order Raman modes. Given that the first-order modes in 1L-WSe_2_ are accidentally degenerate, the more sensitive higher-order Raman bands stand out as a more efficient strain indicator.

Our work sheds light on the strain transfer effects in 2D material based devices and highlights Raman spectroscopy as a versatile tool for the nondestructive strain sensing in 2D material flexible electronics. The strain dependence of the higher-order Raman modes sheds light to the intricate electron phonon coupling and resonance Raman scattering effects in 2D WSe_2_. Future work involves characterizing electrical device characteristics under different types of strain, such as uniaxial, and investigating the influence of interfacial defects and encapsulation layers on the strain response of TMD materials.

## Supporting Information Available

The determination of the strain transfer efficiency from PMMA to PEN; the strain dependence of additional MoS_2_ transistors; correlation plots and strain maps for the rest of the investigated devices; transfer curves for representative WSe_2_ devices; the WSe_2_/PEN and WSe_2_/AlO_*x*_/PEN samples; detail of the WSe_2_ Raman spectrum near 260 cm^–1^; measurements with the high-resolution Raman spectrometer; details on the calculation of strain transfer efficiency based on strain transfer theory.(PDF)

## References

[ref1] Mak K. F.; Lee C.; Hone J.; Shan J.; Heinz T. F. Atomically Thin MoS_2_: A New Direct-Gap Semiconductor. Phys. Rev. Lett. 2010, 105 (13), 136805 10.1103/PhysRevLett.105.136805. 21230799

[ref2] Splendiani A.; Sun L.; Zhang Y.; Li T.; Kim J.; Chim C.-Y.; Galli G.; Wang F. Emerging Photoluminescence in Monolayer MoS_2_. Nano Lett. 2010, 10 (4), 1271–1275. 10.1021/nl903868w. 20229981

[ref3] Mak K. F.; He K.; Lee C.; Lee G. H.; Hone J.; Heinz T. F.; Shan J. Tightly Bound Trions in Monolayer MoS_2_. Nat. Mater. 2013, 12 (3), 207–211. 10.1038/nmat3505. 23202371

[ref4] Lin K.-Q. A Roadmap for Interlayer Excitons. Light Sci. Appl. 2021, 10 (1), 99 10.1038/s41377-021-00544-3. 33966041 PMC8106673

[ref5] Lui C. H.; Frenzel A. J.; Pilon D. V.; Lee Y.-H.; Ling X.; Akselrod G. M.; Kong J.; Gedik N. Trion-Induced Negative Photoconductivity in Monolayer MoS_2_. Phys. Rev. Lett. 2014, 113 (16), 166801 10.1103/PhysRevLett.113.166801. 25361273

[ref6] Novoselov K. S.; Geim A. K.; Morozov S. V.; Jiang D.; Katsnelson M. I.; Grigorieva I. V.; Dubonos S. V.; Firsov A. A. Two-Dimensional Gas of Massless Dirac Fermions in Graphene. Nature 2005, 438 (7065), 197–200. 10.1038/nature04233. 16281030

[ref7] Fu Q.; Wang W.; Yang L.; Huang J.; Zhang J.; Xiang B. Controllable Synthesis of High Quality Monolayer WS_2_ on a SiO_2_/Si Substrate by Chemical Vapor Deposition. RSC Adv. 2015, 5 (21), 15795–15799. 10.1039/C5RA00210A.

[ref8] Michail A.; Parthenios J.; Anestopoulos D.; Galiotis C.; Christian M.; Ortolani L.; Morandi V.; Papagelis K. Controllable, Eco-Friendly, Synthesis of Highly Crystalline 2D-MoS_2_ and Clarification of the Role of Growth-Induced Strain. 2D Mater. 2018, 5 (3), 035035 10.1088/2053-1583/aac610.

[ref9] Kim T. S.; Dhakal K. P.; Park E.; Noh G.; Chai H.-J.; Kim Y.; Oh S.; Kang M.; Park J.; Kim J.; Kim S.; Jeong H. Y.; Bang S.; Kwak J. Y.; Kim J.; Kang K. Gas-Phase Alkali Metal-Assisted MOCVD Growth of 2D Transition Metal Dichalcogenides for Large-Scale Precise Nucleation Control. Small 2022, 18 (20), 2106368 10.1002/smll.202106368. 35451163

[ref10] Radisavljevic B.; Radenovic A.; Brivio J.; Giacometti V.; Kis A. Single-Layer MoS_2_ Transistors. Nat. Nanotechnol. 2011, 6 (3), 147–150. 10.1038/nnano.2010.279. 21278752

[ref11] Sun X.; Xu L.; Zhang Y.; Wang W.; Liu S.; Yang C.; Zhang Z.; Lu J. Performance Limit of Monolayer WSe2 Transistors; Significantly Outperform Their MoS_2_ Counterpart. ACS Appl. Mater. Interfaces 2020, 12 (18), 20633–20644. 10.1021/acsami.0c01750. 32285659

[ref12] Daus A.; Vaziri S.; Chen V.; Köroğlu Ç.; Grady R. W.; Bailey C. S.; Lee H. R.; Schauble K.; Brenner K.; Pop E. High-Performance Flexible Nanoscale Transistors Based on Transition Metal Dichalcogenides. Nat. Electron. 2021, 4 (7), 495–501. 10.1038/s41928-021-00598-6.

[ref13] Conley H. J.; Wang B.; Ziegler J. I.; Haglund R. F.; Pantelides S. T.; Bolotin K. I. Bandgap Engineering of Strained Monolayer and Bilayer MoS_2_. Nano Lett. 2013, 13 (8), 3626–3630. 10.1021/nl4014748. 23819588

[ref14] Amin B.; Kaloni T. P.; Schwingenschlögl U. Strain Engineering of WS_2_, WSe_2_, and WTe_2_. RSC Adv. 2014, 4 (65), 34561–34565. 10.1039/C4RA06378C.

[ref15] De Palma A. C.; Cossio G.; Jones K.; Quan J.; Li X.; Yu E. T. Strain-Dependent Luminescence and Piezoelectricity in Monolayer Transition Metal Dichalcogenides. J. Vac. Sci. Technol. B 2020, 38 (4), 042205 10.1116/6.0000251.

[ref16] Rezk A. R.; Carey B.; Chrimes A. F.; Lau D. W. M.; Gibson B. C.; Zheng C.; Fuhrer M. S.; Yeo L. Y.; Kalantar-zadeh K. Acoustically-Driven Trion and Exciton Modulation in Piezoelectric Two-Dimensional MoS_2_. Nano Lett. 2016, 16 (2), 849–855. 10.1021/acs.nanolett.5b02826. 26729449

[ref17] Manzeli S.; Allain A.; Ghadimi A.; Kis A. Piezoresistivity and Strain-Induced Band Gap Tuning in Atomically Thin MoS_2_. Nano Lett. 2015, 15 (8), 5330–5335. 10.1021/acs.nanolett.5b01689. 26191965

[ref18] Nakamura K. First-Principles Simulation of Piezoresistivity of Transition Metal Dichalcogenide Monolayers. Sens. Mater. 2018, 30 (9), 2073–2083. 10.18494/SAM.2018.1957.

[ref19] Moon H.; Grosso G.; Chakraborty C.; Peng C.; Taniguchi T.; Watanabe K.; Englund D. Dynamic Exciton Funneling by Local Strain Control in a Monolayer Semiconductor. Nano Lett. 2020, 20 (9), 6791–6797. 10.1021/acs.nanolett.0c02757. 32790415

[ref20] Feng J.; Qian X.; Huang C.-W.; Li J. Strain-Engineered Artificial Atom as a Broad-Spectrum Solar Energy Funnel. Nat. Photonics 2012, 6 (12), 866–872. 10.1038/nphoton.2012.285.

[ref21] Li H.; Contryman A. W.; Qian X.; Ardakani S. M.; Gong Y.; Wang X.; Weisse J. M.; Lee C. H.; Zhao J.; Ajayan P. M.; Li J.; Manoharan H. C.; Zheng X. Optoelectronic Crystal of Artificial Atoms in Strain-Textured Molybdenum Disulphide. Nat. Commun. 2015, 6 (1), 7381 10.1038/ncomms8381. 26088550 PMC4557352

[ref22] Chen Y.; Deng W.; Chen X.; Wu Y.; Shi J.; Zheng J.; Chu F.; Liu B.; An B.; You C.; Jiao L.; Liu X.; Zhang Y. Carrier Mobility Tuning of MoS_2_ by Strain Engineering in CVD Growth Process. Nano Res. 2020, 2314 10.1007/s12274-020-3228-4.

[ref23] Datye I. M.; Daus A.; Grady R. W.; Brenner K.; Vaziri S.; Pop E. Strain-Enhanced Mobility of Monolayer MoS_2_. Nano Lett. 2022, 22 (20), 8052–8059. 10.1021/acs.nanolett.2c01707. 36198070

[ref24] Yang J. A.; Bennett R. K. A.; Hoang L.; Zhang Z.; Thompson K. J.; Michail A.; Parthenios J.; Papagelis K.; Mannix A. J.; Pop E. Biaxial Tensile Strain Enhances Electron Mobility of Monolayer Transition Metal Dichalcogenides. ACS Nano 2024, 18, 18151 10.1021/acsnano.3c08996. 38921699

[ref25] Rice C.; Young R. J.; Zan R.; Bangert U.; Wolverson D.; Georgiou T.; Jalil R.; Novoselov K. S. Raman-Scattering Measurements and First-Principles Calculations of Strain-Induced Phonon Shifts in Monolayer MoS_2_. Phys. Rev. B 2013, 87 (8), 081307 10.1103/PhysRevB.87.081307.

[ref26] Mohiuddin T. M. G.; Lombardo A.; Nair R. R.; Bonetti A.; Savini G.; Jalil R.; Bonini N.; Basko D. M.; Galiotis C.; Marzari N.; Novoselov K. S.; Geim A. K.; Ferrari A. C. Uniaxial Strain in Graphene by Raman Spectroscopy: G Peak Splitting, Grüneisen Parameters, and Sample Orientation. Phys. Rev. B 2009, 79 (20), 205433 10.1103/PhysRevB.79.205433.

[ref27] Androulidakis C.; Koukaras E. N.; Parthenios J.; Kalosakas G.; Papagelis K.; Galiotis C. Graphene Flakes under Controlled Biaxial Deformation. Sci. Rep. 2016, 5 (1), 18219 10.1038/srep18219. PMC4678326 26666692

[ref28] Michail A.; Anestopoulos D.; Delikoukos N.; Parthenios J.; Grammatikopoulos S.; Tsirkas S. A.; Lathiotakis N. N.; Frank O.; Filintoglou K.; Papagelis K. Biaxial Strain Engineering of CVD and Exfoliated Single- and Bi-Layer MoS_2_ Crystals. 2D Mater. 2021, 8 (1), 015023 10.1088/2053-1583/abc2de.

[ref29] Michail A.; Anestopoulos D.; Delikoukos N.; Grammatikopoulos S.; Tsirkas S. A.; Lathiotakis N. N.; Frank O.; Filintoglou K.; Parthenios J.; Papagelis K. Tuning the Photoluminescence and Raman Response of Single-Layer WS_2_ Crystals Using Biaxial Strain. J. Phys. Chem. C 2023, 127 (7), 3506–3515. 10.1021/acs.jpcc.2c06933.

[ref30] Lloyd D.; Liu X.; Christopher J. W.; Cantley L.; Wadehra A.; Kim B. L.; Goldberg B. B.; Swan A. K.; Bunch J. S. Band Gap Engineering with Ultralarge Biaxial Strains in Suspended Monolayer MoS_2_. Nano Lett. 2016, 16 (9), 5836–5841. 10.1021/acs.nanolett.6b02615. 27509768

[ref31] Choudhary M.; Shital S.; Ya’akobovitz A.; Niv A. Shear Strain Bandgap Tuning of Monolayer MoS_2_. Appl. Phys. Lett. 2020, 117 (22), 223102 10.1063/5.0022908.

[ref32] Cao K.; Feng S.; Han Y.; Gao L.; Hue Ly T.; Xu Z.; Lu Y. Elastic Straining of Free-Standing Monolayer Graphene. Nat. Commun. 2020, 11 (1), 284 10.1038/s41467-019-14130-0. 31941941 PMC6962388

[ref33] Mennel L.; Paur M.; Mueller T. Second Harmonic Generation in Strained Transition Metal Dichalcogenide Monolayers: MoS_2_, MoSe_2_, WS_2_, and WSe_2_. APL Photonics 2019, 4 (3), 034404 10.1063/1.5051965.

[ref34] Li Z.; Lv Y.; Ren L.; Li J.; Kong L.; Zeng Y.; Tao Q.; Wu R.; Ma H.; Zhao B.; Wang D.; Dang W.; Chen K.; Liao L.; Duan X.; Duan X.; Liu Y. Efficient Strain Modulation of 2D Materials via Polymer Encapsulation. Nat. Commun. 2020, 11 (1), 1151 10.1038/s41467-020-15023-3. 32123176 PMC7052151

[ref35] Li Y.; Hu Z.; Lin S.; Lai S. K.; Ji W.; Lau S. P. Giant Anisotropic Raman Response of Encapsulated Ultrathin Black Phosphorus by Uniaxial Strain. Adv. Funct. Mater. 2017, 27 (19), 1600986 10.1002/adfm.201600986.

[ref36] Huang Y.; Wang X.; Zhang X.; Chen X.; Li B.; Wang B.; Huang M.; Zhu C.; Zhang X.; Bacsa W. S.; Ding F.; Ruoff R. S. Raman Spectral Band Oscillations in Large Graphene Bubbles. Phys. Rev. Lett. 2018, 120 (18), 186104 10.1103/PhysRevLett.120.186104. 29775365

[ref37] Tyurnina A. V.; Bandurin D. A.; Khestanova E.; Kravets V. G.; Koperski M.; Guinea F.; Grigorenko A. N.; Geim A. K.; Grigorieva I. V. Strained Bubbles in van Der Waals Heterostructures as Local Emitters of Photoluminescence with Adjustable Wavelength. ACS Photonics 2019, 6 (2), 516–524. 10.1021/acsphotonics.8b01497.

[ref38] Chen L.; Xue F.; Li X.; Huang X.; Wang L.; Kou J.; Wang Z. L. Strain-Gated Field Effect Transistor of a MoS_2_–ZnO 2D–1D Hybrid Structure. ACS Nano 2016, 10 (1), 1546–1551. 10.1021/acsnano.5b07121. 26695840

[ref39] Ryu Y. K.; Carrascoso F.; López-Nebreda R.; Agraït N.; Frisenda R.; Castellanos-Gomez A. Microheater Actuators as a Versatile Platform for Strain Engineering in 2D Materials. Nano Lett. 2020, 20 (7), 5339–5345. 10.1021/acs.nanolett.0c01706. 32491864

[ref40] Plechinger G.; Castellanos-Gomez A.; Buscema M.; van der Zant H. S. J.; Steele G. A.; Kuc A.; Heine T.; Schüller C.; Korn T. Control of Biaxial Strain in Single-Layer Molybdenite Using Local Thermal Expansion of the Substrate. 2D Mater. 2015, 2 (1), 015006 10.1088/2053-1583/2/1/015006.

[ref41] Zhao W.; Ghorannevis Z.; Chu L.; Toh M.; Kloc C.; Tan P.-H.; Eda G. Evolution of Electronic Structure in Atomically Thin Sheets of WS_2_ and WSe_2_. ACS Nano 2013, 7 (1), 791–797. 10.1021/nn305275h. 23256505

[ref42] Martin R. M.; Falicov L. M. Resonance Raman Scattering. In Light Scattering in Solids I; Cardona M., Ed.; Springer-Verlag, 1983; Vol. 8, p 79.

[ref43] Carvalho B. R.; Pimenta M. A. Resonance Raman Spectroscopy in Semiconducting Transition-Metal Dichalcogenides: Basic Properties and Perspectives. 2D Mater. 2020, 7 (4), 042001 10.1088/2053-1583/ab98ef.

[ref44] Ferrari A. C.; Basko D. M. Raman Spectroscopy as a Versatile Tool for Studying the Properties of Graphene. Nat. Nanotechnol. 2013, 8 (4), 235–246. 10.1038/nnano.2013.46. 23552117

[ref45] Vaziri S.; Yalon E.; Muñoz Rojo M.; Suryavanshi S. V.; Zhang H.; McClellan C. J.; Bailey C. S.; Smithe K. K. H.; Gabourie A. J.; Chen V.; Deshmukh S.; Bendersky L.; Davydov A. V.; Pop E. Ultrahigh Thermal Isolation across Heterogeneously Layered Two-Dimensional Materials. Sci. Adv. 2019, 5 (8), eaax1325 10.1126/sciadv.aax1325. 31453337 PMC6697438

[ref46] International Roadmap for Devices and Systems (IRDS^TM^) 2022 Edition - IEEE IRDS^TM^. https://irds.ieee.org/editions/2022 (accessed July 8, 2024).

[ref47] Urban F.; Giubileo F.; Grillo A.; Iemmo L.; Luongo G.; Passacantando M.; Foller T.; Madauß L.; Pollmann E.; Geller M. P.; Oing D.; Schleberger M.; Bartolomeo A. D. Gas Dependent Hysteresis in MoS_2_ Field Effect Transistors. 2D Mater. 2019, 6 (4), 045049 10.1088/2053-1583/ab4020.

[ref48] Liu X.; Choi M. S.; Hwang E.; Yoo W. J.; Sun J. Fermi Level Pinning Dependent 2D Semiconductor Devices: Challenges and Prospects. Adv. Mater. 2022, 34 (15), 2108425 10.1002/adma.202108425. 34913205

[ref49] Watson A. J.; Lu W.; Guimarães M. H. D.; Stöhr M. Transfer of Large-Scale Two-Dimensional Semiconductors: Challenges and Developments. 2D Mater. 2021, 8 (3), 032001 10.1088/2053-1583/abf234.

[ref50] Johnson P. B.; Christy R. W. Optical Constants of the Noble Metals. Phys. Rev. B 1972, 6 (12), 4370–4379. 10.1103/PhysRevB.6.4370.

[ref51] Zhao W.; Ghorannevis Z.; Amara K. K.; Pang J. R.; Toh M.; Zhang X.; Kloc C.; Tan P. H.; Eda G. Lattice Dynamics in Mono- and Few-Layer Sheets of WS_2_ and WSe_2_. Nanoscale 2013, 5 (20), 9677–9683. 10.1039/c3nr03052k. 23999910

[ref52] Kim S.; Kim K.; Lee J.-U.; Cheong H. Excitonic Resonance Effects and Davydov Splitting in Circularly Polarized Raman Spectra of Few-Layer WSe_2_. 2D Mater. 2017, 4 (4), 045002 10.1088/2053-1583/aa8312.

[ref53] Chen S.-Y.; Zheng C.; Fuhrer M. S.; Yan J. Helicity-Resolved Raman Scattering of MoS_2_, MoSe_2_, WS_2_, and WSe_2_ Atomic Layers. Nano Lett. 2015, 15 (4), 2526–2532. 10.1021/acs.nanolett.5b00092. 25719859

[ref54] Mahrouche F.; Rezouali K.; Mahtout S.; Zaabar F.; Molina-Sánchez A. Phonons in WSe_2_/MoSe_2_ van Der Waals Heterobilayers. Phys. Status Solidi B 2022, 259 (1), 2100321 10.1002/pssb.202100321.

[ref55] del Corro E.; Terrones H.; Elias A.; Fantini C.; Feng S.; Nguyen M. A.; Mallouk T. E.; Terrones M.; Pimenta M. A. Excited Excitonic States in 1L, 2L, 3L, and Bulk WSe_2_ Observed by Resonant Raman Spectroscopy. ACS Nano 2014, 8 (9), 9629–9635. 10.1021/nn504088g. 25162682

[ref56] Berkdemir A.; Gutiérrez H. R.; Botello-Méndez A. R.; Perea-López N.; Elías A. L.; Chia C.-I.; Wang B.; Crespi V. H.; López-Urías F.; Charlier J.-C.; Terrones H.; Terrones M. Identification of Individual and Few Layers of WS_2_ Using Raman Spectroscopy. Sci. Rep. 2013, 3 (1), 1755 10.1038/srep01755.

[ref57] Carvalho B. R.; Wang Y.; Mignuzzi S.; Roy D.; Terrones M.; Fantini C.; Crespi V. H.; Malard L. M.; Pimenta M. A. Intervalley Scattering by Acoustic Phonons in Two-Dimensional MoS_2_ Revealed by Double-Resonance Raman Spectroscopy. Nat. Commun. 2017, 8 (1), 14670 10.1038/ncomms14670. 28276472 PMC5347091

[ref58] Venezuela P.; Lazzeri M.; Mauri F. Theory of Double-Resonant Raman Spectra in Graphene: Intensity and Line Shape of Defect-Induced and Two-Phonon Bands. Phys. Rev. B 2011, 84 (3), 035433 10.1103/PhysRevB.84.035433.

[ref59] Saito R.; Jorio A.; Souza Filho A. G.; Dresselhaus G.; Dresselhaus M. S.; Pimenta M. A. Probing Phonon Dispersion Relations of Graphite by Double Resonance Raman Scattering. Phys. Rev. Lett. 2001, 88 (2), 027401 10.1103/PhysRevLett.88.027401. 11801034

[ref60] Maultzsch J.; Reich S.; Thomsen C. Double-Resonant Raman Scattering in Graphite: Interference Effects, Selection Rules, and Phonon Dispersion. Phys. Rev. B 2004, 70 (15), 155403 10.1103/PhysRevB.70.155403.

[ref61] Ajovalasit A.; Fragapane S.; Zuccarello B. The Reinforcement Effect of Strain Gauges Embedded in Low Modulus Materials. Strain 2013, 49 (4), 366–376. 10.1111/str.12043.

[ref62] Stehlin P. Strain Distribution in and around Strain Gauges. J. Strain Anal. 1972, 7 (3), 228–235. 10.1243/03093247V073228.

