# Supplementary material for: Biaxial Strain Transfer in Monolayer MoS2 and WSe2 Transistor Structures
Source: ACS Appl Mater Interfaces. 2024 Sep 3;16(37):49602–11. doi: 10.1021/acsami.4c07216 (PMC11420877; doi:10.1021/acsami.4c07216)
Supplement: Supplementary file 1 — am4c07216_si_001.pdf [file am4c07216_si_001.pdf]

Supporting information for Biaxial strain transfer in monolayer MoS_2 and WSe_2 transistor structures

Antonios Michail[1,2], Jerry A. Yang[3], Kyriakos Filintoglou[4], Nikolaos Balakeras[4], Crystal Alicia Nattoo[3], Connor Scott Bailey[3], Alwin Daus[3,5], John Parthenios[*,2], Eric Pop[3,6,7], and Konstantinos Papagelis[*,2,4]

[1]Department of Physics, University of Patras, Patras 26504, Greece
[2]Institute of Chemical Engineering Sciences, Foundation for Research and Technology Hellas (FORTH – ICE/HT), Patras 26504, Greece
[3]Department of Electrical Engineering, Stanford University, Stanford, California 94305, USA
[4]School of Physics, Department of Solid State Physics, Aristotle University of Thessaloniki, Thessaloniki 54124, Greece
[5]Department of Microsystems Engineering, University of Freiburg, Freiburg 79110, Germany
[6]Department of Materials Science, Stanford University, Stanford, California 94305, USA
[7]Precourt Institute for Energy, Stanford University, Stanford, CA, 94305, USA
[*]Corresponding author e-mail: jparthen@iceht.forth.gr, kpapag@physics.auth.gr

Section 1. Determination of the strain transfer efficiency from PMMA to PEN

Attachment of the PEN film on the PMMA cruciform was accomplished by first cleaning the PMMA surface with isopropanol and applying a droplet of cyanoacrylate adhesive (CA) on its center, as shown in Figure S1 (a). Immediately after CA deposition the PEN film was positioned carefully on the still liquid CA droplet. The liquid CA now expands and increases the contact area with PEN film (Figure S1 (b)). Immediately, a nitrogen gun nozzle is positioned perpendicularly and very close to the PEN film, roughly 2 mm above it. The N_2 gun is triggered, and the nitrogen flow presses the PEN film uniformly on the PMMA substrate while excess CA is pushed out of the interface Figure S1(c). The resulting adhesion layer after full curing of the CA (24 hours curing time) was measured to be approximately 20 μm. Note that this way, no actual contact is made with the devices on top of the PEN film, and subsequent optical microscopic inspection revealed that all devices were intact after attachment of the PEN film to the cruciform.

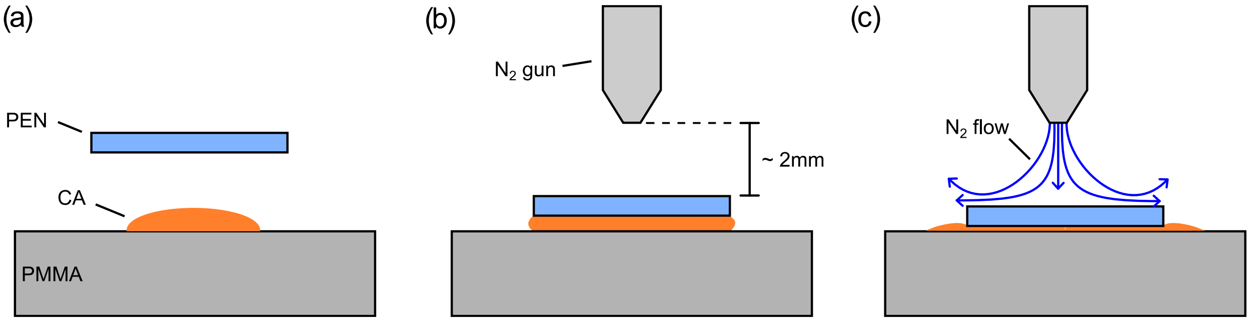

Figure S1 *Attachment of a PEN film to the PMMA cruciform. (a) a drop of cyanoacrylate glue (CA) is placed on the PMMA surface. The PEN film is gently let to sit on the still liquid CA. (b) Upon contact between PEN film and CA, the latter wets almost the whole bottom surface of the former. The nitrogen gun is placed in very close proximity to the PEN film and kept facing perpendicular to the PEN film. (c) The nitrogen gun is triggered, and the gas flow presses uniformly the PEN film to the PMMA surface pushing outwards excess CA.*

Several adhesives were tested in order to obtain a thin and strong adhesion layer, capable of achieving a very high strain transfer efficiency. Among the materials used, cyanoacrylate adhesive (Logo™ instant glue) was

found to possess the desired properties of easy handling, formation of a thin adhesive layer (approximately 20 µm) and high strain transfer efficiency of 87 %.

Assessment of the strain transfer efficiency was made by using linear resistive foil strain gauges (KYOWA KFP-5-120-C1-65). One gauge was attached directly on a PMMA cruciform while identical strain gauges were attached on the PEN films that were previously bonded to cruciforms using the tested adhesives. The gauge resistance is related to the strain components in the parallel and normal direction of the gauge axis as [1]:

$$R(\delta) - R_o = R_o(F_a \varepsilon_\alpha + F_t \varepsilon_t) \quad \text{(S1)}$$

where F_a, F_t are the axial and transverse gauge factors and ε_α, ε_t, are the axial and transverse (to the gauge axis) strain components, respectively. Also, $R(\delta)$ and R_o are the gauge resistance at finite and zero deflection δ, respectively. Note that mechanical strain is applied by increasing the deflection of the cruciform center by a high precision screw (see supporting information in ref. [2] for extensive details of the strain device). The strain components are proportional to δ, and due to the applied strain being biaxial ($\varepsilon_a = \varepsilon_\tau = \varepsilon$), the rate of change of the gauge resistance with respect to δ is:

$$R' = R_o(F_a + F_t)\varepsilon' \quad \text{(S2)}$$

where $R' = \frac{dR}{d\delta}$ and $\varepsilon' = \frac{d\varepsilon}{d\delta}$. Finally, the strain transfer efficiency for identical gauges can be determined by taking the ratio:

$$\frac{R'_{PEN}}{R'_{REF}} = \frac{\varepsilon'_{PEN}}{\varepsilon'_{REF}} \quad \text{(S3)}$$

where the subscripts PEN and REF refer to the gauges attached to the bonded PEN and the reference PMMA cruciform, respectively. Figure S2 shows the change of the gauge resistance as a function of applied deflection, δ, for two samples attached using cyanoacrylate adhesive (blue and black) and the reference sample (red). The obtained slopes were 0.146(2), 0.145(2) and 0.168(4) Ω/mm for CA1, CA2 and reference samples, respectively. Thus, the average strain transfer efficiency is determined at 0.87. Also, in the same figure it is evident that linearity is preserved for all samples at least up to the measured displacement of 8 mm, which in our device corresponds to a nominal strain of about 0.70 % on a 3 mm thick PMMA cruciform or to approximately 0.61 % at the bonded PEN film.

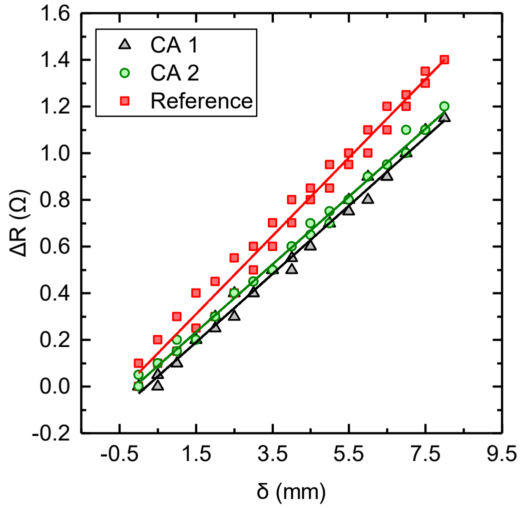

Figure S2 *The change of the strain gauge resistance as a function of central displacement, δ, for two PEN films bonded to PMMA, labeled CA 1 (black triangles) and CA2 (green circles), and a bare PMMA reference sample (red squares). The ratio of the average slope of the CA1 and CA2 lines to the slope of the reference sample line is 0.87.*

Section 2. Strain dependence of the Raman modes of additional MoS_2 transistors

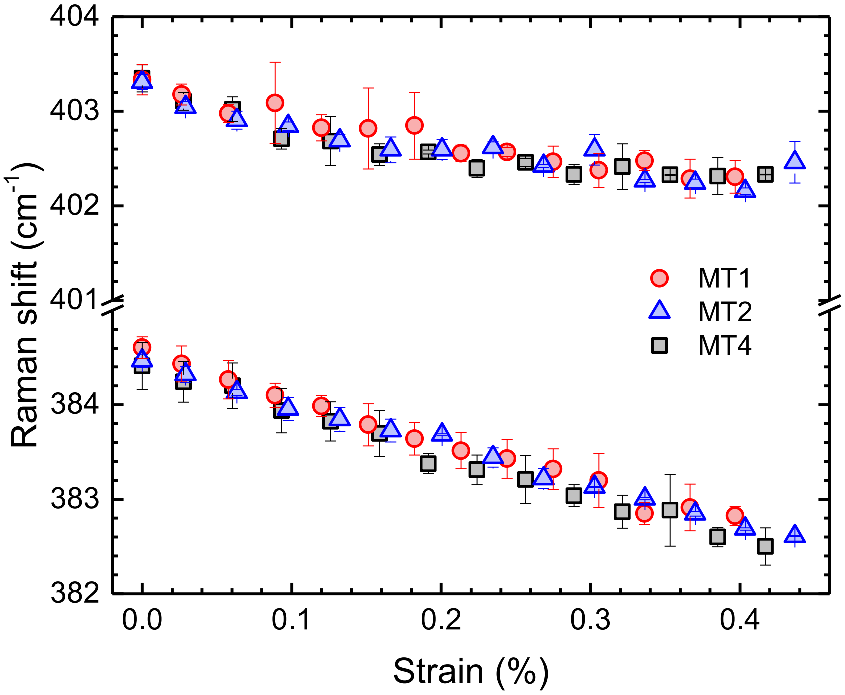

Figure S3 *Plot of the Raman peak position vs strain for devices MT1 (red circles), MT2 (blue triangles) and MT4 (black squares).*

Section 3. Correlation plots and strain maps for the rest of the investigated MoS_2 devices

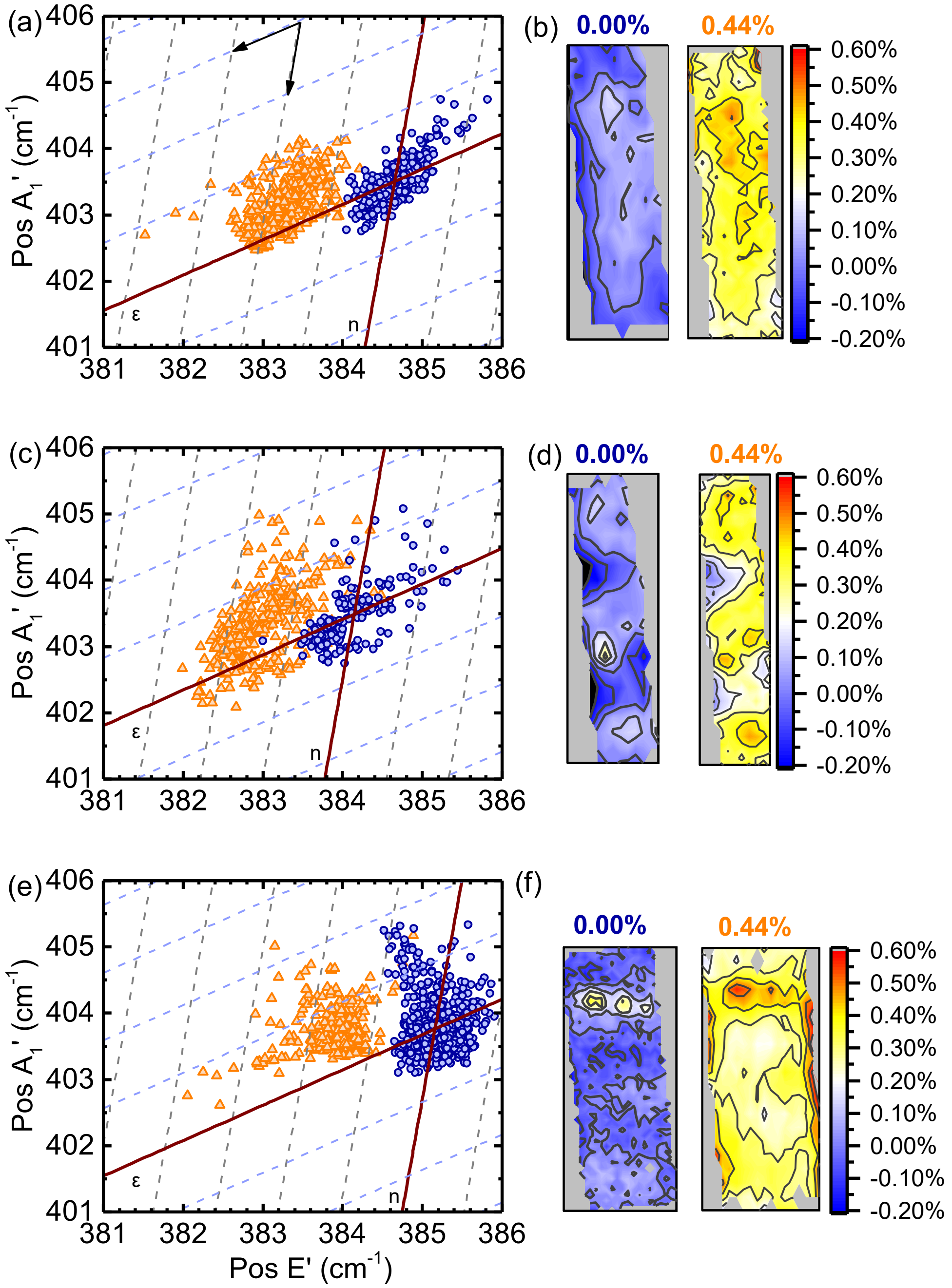

Figure S4 *(a), (c), (e): Pos*(A_1') *vs Pos*(E') *correlation plots for the three additional devices (MT2, MT3 and MT4) that were studied. (b), (d) and (f) show the corresponding strain maps at zero and maximum applied strains.*

Section 4. Transfer curves for representative WSe_2 devices

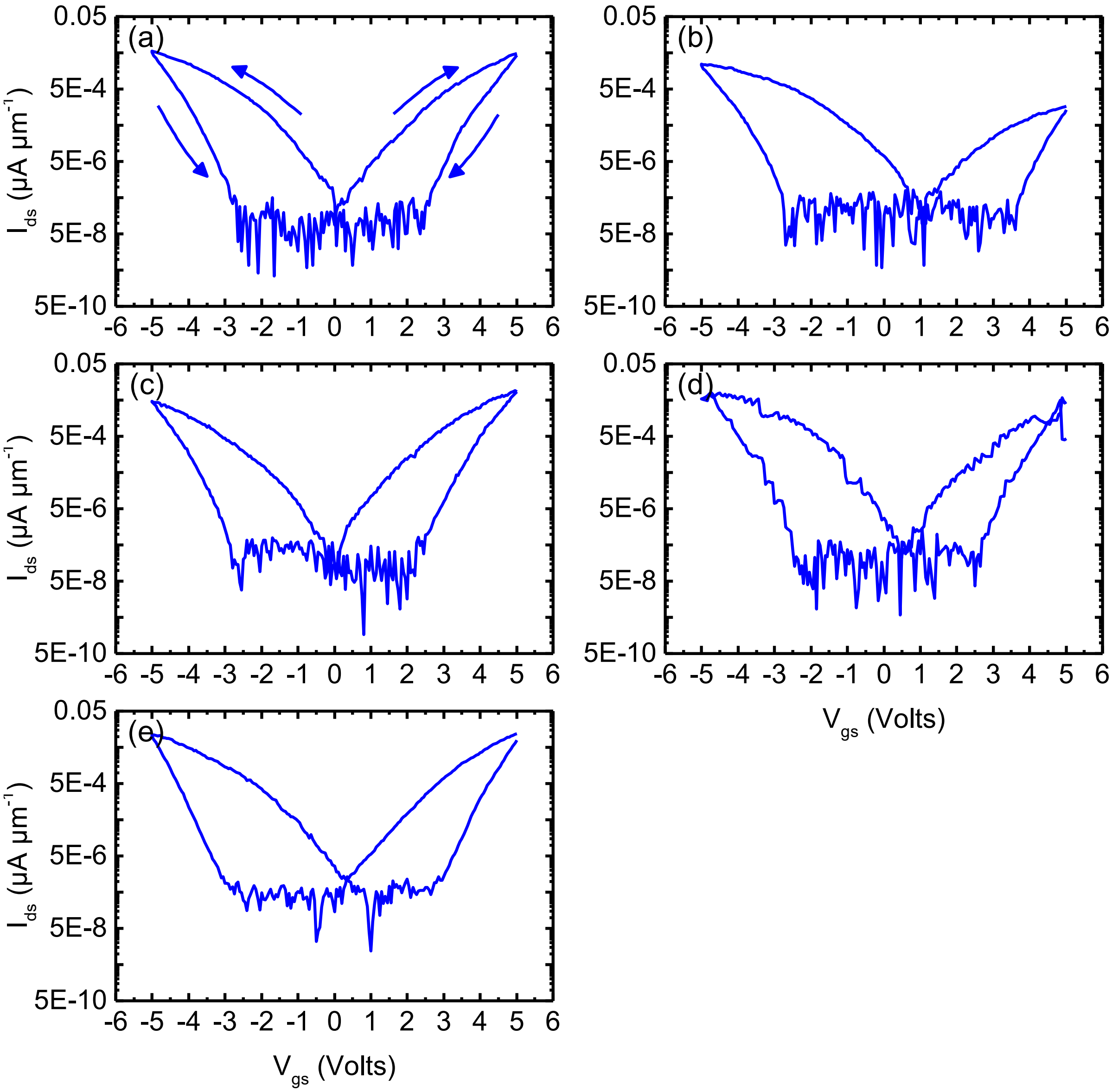

Figure S5 *(a) –(e) Transfer curves for representative WSe_2 devices at zero strain. Semiconducting behavior and ambipolarity is observed, confirming that the quality of our CVD WSe_2 is sufficient for field effect transistor operation. The arrows in (a) show the sweep direction for all plots.*

Section 5. The WSe_2/PEN and WSe_2/AlO_x/PEN samples

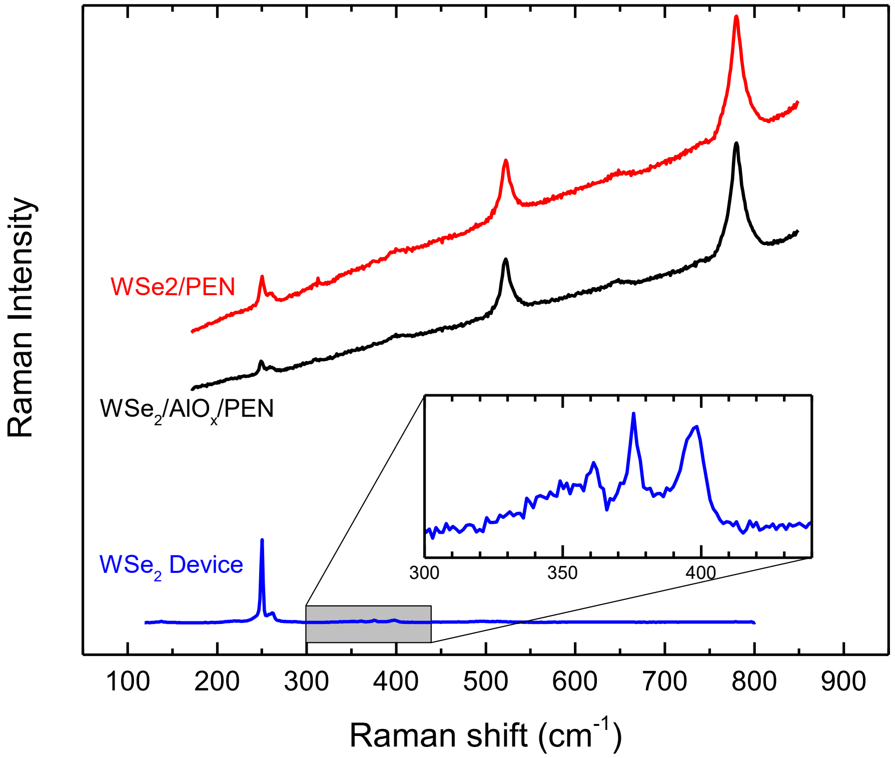

Figure S6 *Comparison of Raman spectra excited with λ = 515 nm from 1L - WSe_2/PEN film (red),1L - WSe_2 /AlO_x /PEN film (black) and the channel region of a WSe_2 transistor (blue). The exposure time and laser intensity are different for each case to maximize spectrum quality. The inset shows the 300 - 440 cm^{-1}region of the transistor spectrum where the WSe_2 second order Raman peaks are detected. Those peaks are not detected in the other spectra due to background luminescence.*

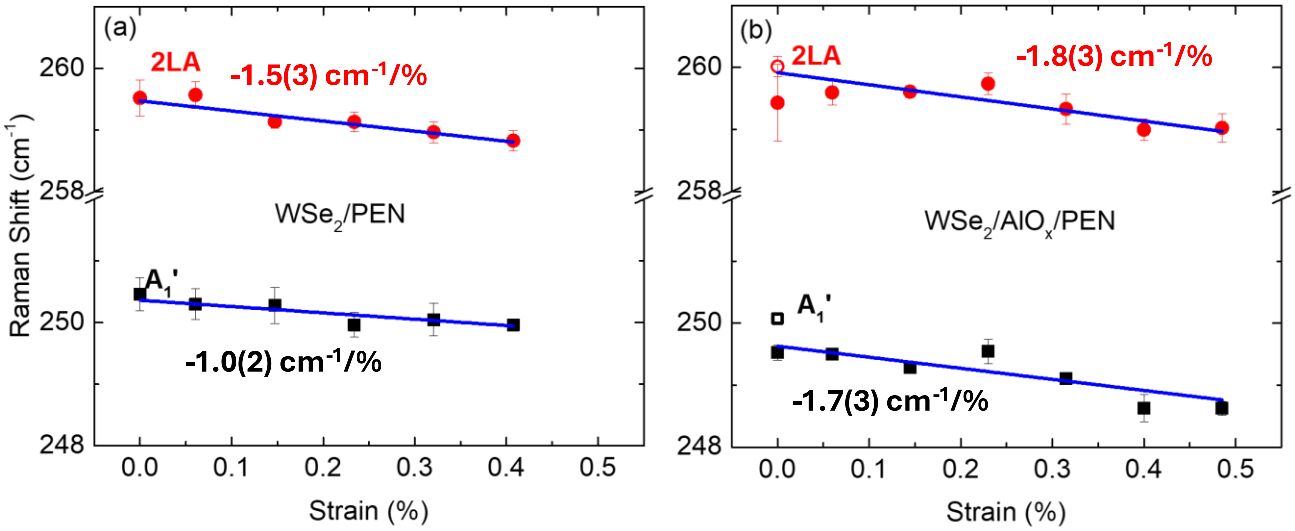

Figure S7 *Strain dependence of the A_1' and $2LA$ Raman peaks for (a) the WSe_2/PEN and (b) the WSe_2/AlO_x/PEN samples.*

Section 6. Detail of the WSe_2 Raman spectrum near 260 cm^{-1}

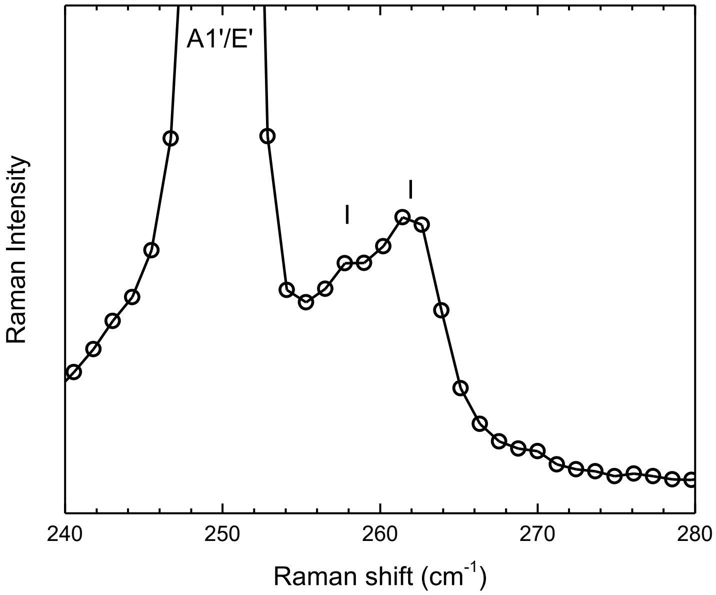

Figure S8 *Detail of the Raman spectrum of 1L-WSe₂ showing the feature on the high frequency side of the* A_1'/E' *peak attributed to contributions from the* $2LA$ *and* $A'(M)$ *peak. The spectrum was collected from a* WSe_2 *transistor with* $\lambda_{exc} = 515$ *nm.*

Section 7. Measurements with the high resolution spectrometer DILOR XY (514.5 nm)

Table S-I Peak frequencies at zero strain and shift rates obtained with 514.5 nm excitation.

**Peak**	ω_o **(cm^{-1})**	$\frac{d\omega}{d\varepsilon}$ **(cm^{-1}/%)**
A_1'	248.2	-1.4(5)
$2LA$	256.6	-3.1(6)
$A(M)$	260.4	-3.5(3)
p_1	357.1	-6(1)
p_2	372.9	-4.3(4)
p_3/p_4	395.3	-3(1)

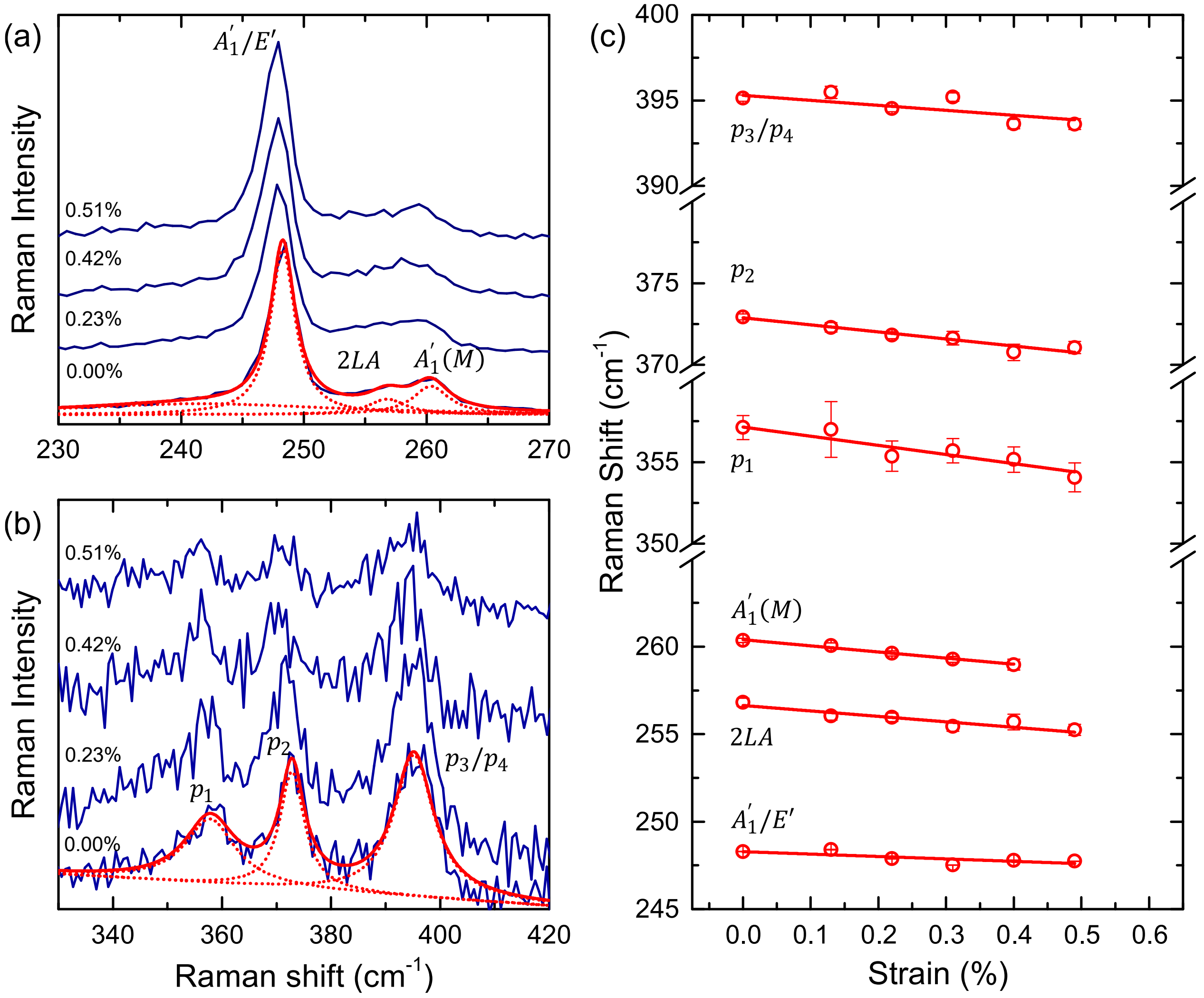

Figure S9 *Raman spectra of WSe_2 under 514.5 nm excitation at different strain levels for (a) the $A_1'/2LA$ peak region and (b) the higher order peaks between 340 – 420 cm^{-1}. The red solid lines are the total fit curve while the dashed lines show the individual Lorentzian components. (c) The strain dependence of the detected peaks.*

Section 8. Strain transfer theory

The structure of the devices used in this work, i.e., polymer film - metal - Gate oxide - 2D material, is similar to the structure of a resistive foil strain gauge bonded to a low modulus material. Indeed, typical strain gauges are usually made of a thin metallic grid that is supported or encapsulated on a flexible polymer film and is usually bonded with a cyanoacrylate adhesive to the test specimen [3]. A problem arises when the gauge is bonded to low modulus material such as plastics. If the specimen is more compliant than the strain gauge a reinforcement effect occurs [4]. This affects the strain indicated by the gauge which can differ significantly from the true strain imposed on a specimen without an attached gauge.

The problem of determining the strain transfer efficiency in strain sensors is of significant importance in the field of engineering and has been studied extensively in the past [4–8]. Stehlin [5], developed a simplified 2D model to study the strain distribution in and around strain gauges, bonded on the surface of a substrate. In his model the substrate was treated as a semi-infinite space, while the gauge was treated as thin strip and an adhesive layer was considered between gauge and substrate. By making some simplifying assumptions he derived an

integrodifferential equation which can be solved numerically to obtain the strain transfer efficiency along the length of the strain gauge. Using the same methodology, it can be shown that in the case of many intermediate layers instead of just one adhesive layer, Stehlin's integrodifferential equation still holds with minor change of the β parameter (see eq. (S4)).

$$1-\frac{\alpha}{\pi}\int_{-1}^{1}\frac{\dot{\eta}(\xi')d\xi'}{\xi-\xi'}+\beta\ddot{\eta}(\xi)-\eta(\xi)=0 \quad \text{(S4)}$$

Here, the parameters a and β are define as $a=\frac{4t_{top}E_{top}}{E_{sub}L}$, $\beta=\frac{4A\,t_{top}E_{top}}{L^2}$ and $A=\sum_{i=1}^{N}\frac{t_i}{G_i}$ with t, E and G being the thickness, Young's modulus and shear modulus of a layer, respectively. The layer is indicated by the appropriate subscript, i.e., "top" for top layer, "sub" for substrate or $i=1,2,3,\dots,N$ for the intermediate layers. Note that the sum in A, runs over intermediate layers only (substrate and top are excluded). Finally, L is the channel width, $\eta=\frac{\varepsilon_{top}}{\varepsilon_{sub}}$ is the strain transfer efficiency from substrate to the top layer, and $\xi=\frac{x}{L/2}$ is the non-dimensional coordinate. A dot over a symbol indicates differentiation, i.e., $\dot{\eta}=\frac{d\eta}{d\xi}$ and $\ddot{\eta}=\frac{d^2\eta}{d\xi^2}$. The values and definition of all parameters are shown in Table S-II, and Figure S10 introduces the relevant geometry. If only one intermediate layer is considered (with thickness t_L and shear modulus G_L), then equation (S4) reverts back to the original form presented in [5].

As mentioned earlier, the applied strain in the PEN film supporting the devices was established experimentally to be around 0.87 %. Solving eq. (S4) numerically using the collocation method (see appendix in reference [5]), and considering PEN as substrate, Ti (5nm) – Gold (40 nm) and AlOx (18 nm) as intermediate layers, and 1L MoS_2 (0.7 nm) as a top layer, the strain transfer efficiency at the center of the channel (ξ = 0) as a function of channel length, L, was calculated. As shown in Figure S11, for $L=1$ µm the efficiency is about 90%, while for the channel dimensions used in this work (5 µm) the efficiency is 98%. This supports the results presented in the manuscript.

Another interesting observation can be made by applying this methodology to the stack PMMA (substrate) – Cyanoacrylate adhesive (20 µm) – PEN (125 µm), by setting the parameters the following values for the parameters α,β:

$$a=\frac{4t_{PEN}E_{PEN}}{E_{PMMA}L_{PEN}} \quad \text{(S5)}$$

$$\beta=\frac{4t_{CA}\,t_{PEN}E_{MoS_2}}{G_{CA}L^2} \quad \text{(S6)}$$

In this case, the determined strain transfer efficiency was 0.94 %, slightly larger than the 0.87% which was measured experimentally. Considering the simplified treatment and errors involved in the elastic moduli of the materials, this discrepancy is more than acceptable and provides further theoretical support for our observations.

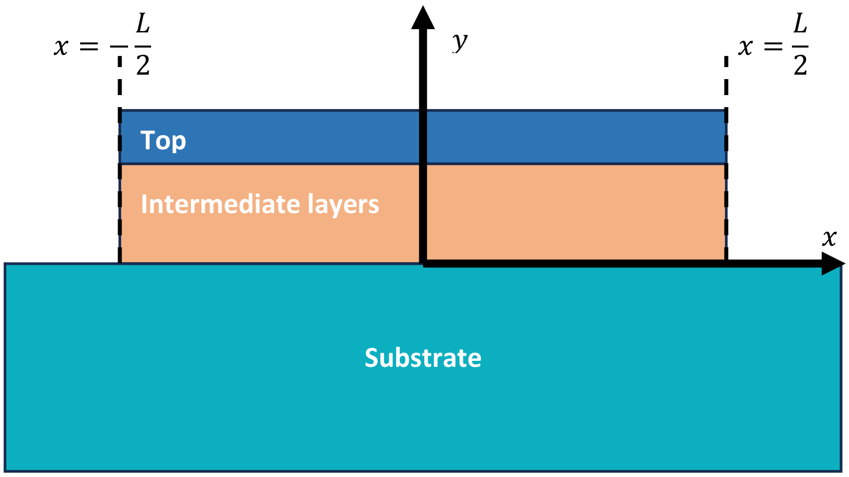

***Figure S10** The geometry of the problem. The substrate is a half-space that experiences strain $\varepsilon_{xx} = \varepsilon_{sub}$. The strain is transferred through the intermediate layers only through shear stresses. The intermediate layers have length L. Equation (S4) gives the distribution of strain on the top strip along its length.*

Table S-II Parameter symbols, values and definitions used in calculating the solution of eq. (4)

Symbol	Value	Definition
L_{ch}	5 µm	Channel length.
L_{PEN}	12 mm	Lateral dimensions of PEN film
t_{MoS_2}	0.7 nm	MoS_2 thickness.
t_{ox}	18 nm	AlOx thickness.
t_{Au}	40 nm	Gate electrode thickness.
t_{ti}	5 nm	Ti thickness.
t_{PEN}	125 µm	PEN film nominal thickness.
t_{CA}	20 µm	Adhesive thickness.
E_{MoS_2}	300 GPa	Young modulus of 1L-MoS_2 [9,10].
E_{ox}	180 GPa	Tensile modulus of ALD AlOx [11].
E_{Au}	150 GPa	Extrapolated value of the Young modulus of gold evaporated single crystal thin films [12].
E_{Ti}	116 GPa	Young modulus of Titanium [13].
E_{PEN}	5.5 GPa	Young modulus of PEN [14] [15]
E_{PMMA}	2.55 GPa	Young modulus of PMMA [2].
v_{ox}	0.231	Poisson ratio of alumina [16].
v_{Au}	0.42	Poisson ratio of Gold [13].
v_{Ti}	0.36	Posson ratio of Titanium.
v_{PEN}	0.32	Poisson ratio of PEN [17].
G_{ox}	72.6 GPa	Shear modulus of AlOx(a).
G_{Au}	52.8 GPa	Shear modulus of Gold(a).
G_{Ti}	42.6 GPa	Shear modulus of Titanium(a).
G_{CA}	1.3 GPa	Shear modulus of adhesive [5].
G_{PEN}	2.08 GPa	Shear modulus of PEN(a).

(a) Obtained as $G = \frac{E}{2(1+v)}$

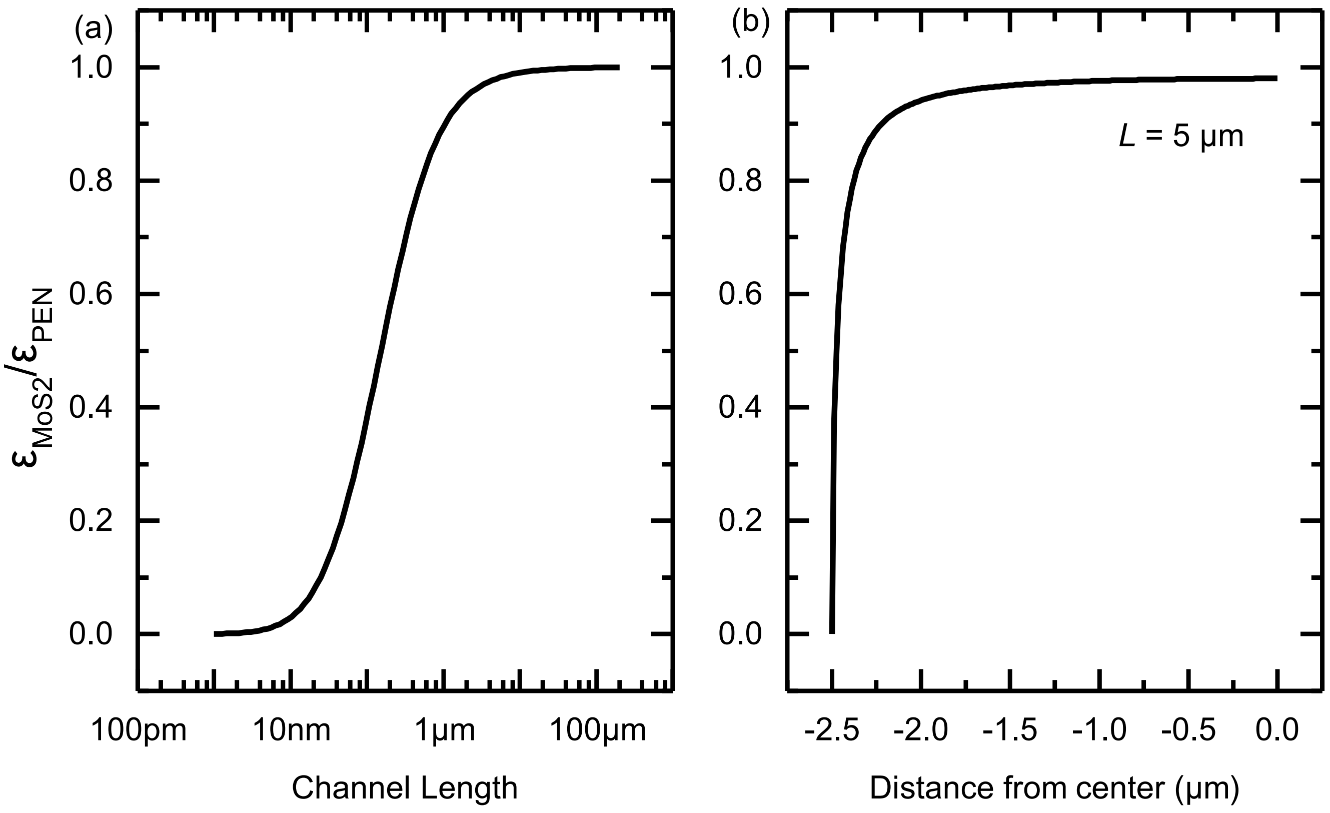

Figure S11 *(a) Strain transfer efficiency at $x = 0$ for a PEN/Ti (5nm)/Au (40 nm)/AlOx (18 nm)/ 1L – MoS_2 stack. (b) Distribution of the strain transfer efficiency over the length of an MoS_2 strip with $L = 5$ µm. The center is at x = 0.*

References

[1] Anon 1983 Errors Due to Transverse Sensitivity in Strain Gages *Experimental Techniques* **7** 30–5

[2] Michail A, Anestopoulos D, Delikoukos N, Grammatikopoulos S, Tsirkas S A, Lathiotakis N N, Frank O, Filintoglou K, Parthenios J and Papagelis K 2023 Tuning the Photoluminescence and Raman Response of Single-Layer WS2 Crystals Using Biaxial Strain *J. Phys. Chem. C* **127** 3506–15

[3] Watson R B 2008 Bonded Electrical Resistance Strain Gages *Springer Handbook of Experimental Solid Mechanics* ed W N Sharpe (Boston, MA: Springer US) pp 283–334

[4] Ajovalasit A, Fragapane S and Zuccarello B 2013 The Reinforcement Effect of Strain Gauges Embedded in Low Modulus Materials *Strain* **49** 366–76

[5] Stehlin P 1972 Strain distribution in and around strain gauges *Journal of Strain Analysis* **7** 228–35

[6] Ajovalasit A and Zuccarello B 2005 Local Reinforcement Effect of a Strain Gauge Installation on Low Modulus Materials *The Journal of Strain Analysis for Engineering Design* **40** 643–53

[7] Ansari F and Libo Y 1998 Mechanics of Bond and Interface Shear Transfer in Optical Fiber Sensors *J. Eng. Mech.* **124** 385–94

[8] Falcetelli F, Rossi L, Di Sante R and Bolognini G 2020 Strain Transfer in Surface-Bonded Optical Fiber Sensors *Sensors* **20** 3100

[9] Bertolazzi S, Brivio J and Kis A 2011 Stretching and breaking of ultrathin MoS_2 *ACS Nano* **5** 9703–9

[10] Castellanos-Gomez A, Poot M, Steele G A, Van Der Zant H S J, Agraït N and Rubio-Bollinger G 2012 Elastic properties of freely suspended MoS_2 nanosheets *Advanced Materials* **24** 772–5

[11] Tripp M K, Stampfer C, Miller D C, Helbling T, Herrmann C F, Hierold C, Gall K, George S M and Bright V M 2006 The mechanical properties of atomic layer deposited alumina for use in micro- and nano-electromechanical systems *Sensors and Actuators A: Physical* **130–131** 419–29

[12] Catlin A and Walker W P 2004 Mechanical Properties of Thin Single-Crystal Gold Films *Journal of Applied Physics* **31** 2135–9

[13] Marks L S 2007 *Marks' standard handbook for mechanical engineers* ed E A Avallone, T Baumeister and A M Sadegh (New York: McGraw-Hill)

[14] Cammarano A, Luca G and Amendola E 2013 Surface modification and adhesion improvement of polyester films *Open Chemistry* **11** 35–45

[15] Fuks L and Degueldre C MATERIALS FOR IN-SITU MONITORING OF LIGHT WATER REACTOR (LWR). WATER CHEMISTRY BY OPTICAL METHODS

[16] Munro M 1997 Evaluated Material Properties for a Sintered alpha-Alumina *Journal of the American Ceramic Society* **80** 1919–28

[17] Ma T, Bhushan B, Murooka H, Kobayashi I and Osawa T 2002 A novel technique to measure the Poisson's ratio and submicron lateral dimensional changes of ultrathin polymeric films *Review of Scientific Instruments* **73** 1813–20
